# Discovery of Novel Bromophenol Hybrids as Potential Anticancer Agents through the Ros-Mediated Apoptotic Pathway: Design, Synthesis and Biological Evaluation

**DOI:** 10.3390/md15110343

**Published:** 2017-11-01

**Authors:** Li-Jun Wang, Chuan-Long Guo, Xiang-Qian Li, Shuai-Yu Wang, Bo Jiang, Yue Zhao, Jiao Luo, Kuo Xu, Hua Liu, Shu-Ju Guo, Ning Wu, Da-Yong Shi

**Affiliations:** 1 Key Laboratory of Experimental Marine Biology, Institute of Oceanology, Chinese Academy of Sciences, Qingdao 266071, Shandong, China; wanglijun@qdio.ac.cn (L.-J.W.); gcl_cpu@126.com (C.-L.G.); lnu101@163.com (X.-Q.L.); 12-12sy@163.com (S.-Y.W.); jiangbo@qdio.ac.cn (B.J.); zhaoyue19931104@163.com (Y.Z.); luojiao2012@163.com (J.L.); xukuoworld@126.com (K.X.); baihualin55100@sina.cn (H.L.); guoshuju@qdio.ac.cn (S.-J.G.); wuning@qdio.ac.cn (N.W.); 2 Laboratory for Marine Drugs and Bioproducts, Qingdao National Laboratory for Marine Science and Technology, Qingdao 266071, Shandong, China; 3 University of Chinese Academy of Sciences, Beijing 10049, China

**Keywords:** bromophenol hybrids, anticancer, structure-activity relationships, ROS, apoptotic pathway

## Abstract

A series of bromophenol hybrids with N-containing heterocyclic moieties were designed, and their anticancer activities against a panel of five human cancer cell lines (A549, Bel7402, HepG2, HCT116 and Caco2) using MTT assay in vitro were explored. Among them, thirteen compounds (**17a**, **17b**, **18a**, **19a**, **19b**, **20a**, **20b**, **21a**, **21b**, **22a**, **22b**, **23a**, and **23b**) exhibited significant inhibitory activity against the tested cancer cell lines. The structure-activity relationships (SARs) of bromophenol derivatives were discussed. The promising candidate compound **17a** could induce cell cycle arrest at G0/G1 phase and induce apoptosis in A549 cells, as well as caused DNA fragmentations, morphological changes and ROS generation by the mechanism studies. Furthermore, compound **17a** suppression of Bcl-2 levels (decrease in the expression of the anti-apoptotic proteins Bcl-2 and down-regulation in the expression levels of Bcl-2) in A549 cells were observed, along with activation caspase-3 and PARP, which indicated that compound **17a** induced A549 cells apoptosis in vitro through the ROS-mediated apoptotic pathway. These results might be useful for bromophenol derivatives to be explored and developed as novel anticancer drugs.

## 1. Introduction

The development of anticancer agents is the hot topic in medicinal chemistry based on the high incidence and lethality of cancer today. Although many effective drugs have been approved to treat cancer, therapies for it are less satisfactory due to side effects, thus there is need for novel therapeutic agents. Certain new targeted agents can block the growth of cancer cells by interfering with specific targeted molecules and have less toxicity than chemotherapy drugs, which offer a promise of treating cancer. Reactive oxygen species (ROS) play vital roles in cell growth, which associate with multiple changes in cellular functions (cell proliferation, migration, differentiation, apoptosis, etc.) in cancer cells [1]. Agents targeting ROS-mediated apoptotic pathway have proven to be attractive for cancer therapy [2]. Some natural and synthetic effective anticancer agents targeting ROS-mediated apoptotic pathway were reported [3,4,5].

**Figure 1:**
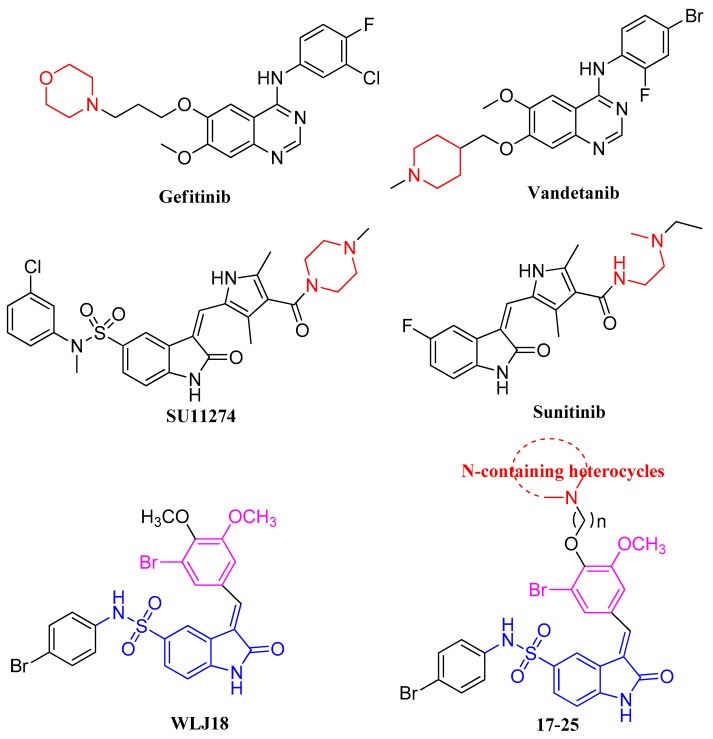
Structures of N-containing heterocyclic anticancer drugs, **WLJ18** and compounds **17**–**25**.

Bromophenols, a class of natural marine products, and their derivatives possessed various potent activities, including antioxidative, anticancer, antithrombotic, antimicrobial, and anti-inflammatory activities, which have attracted much attention [6]. A series of bromophenol hybrids with indolin-2-one moiety were designed and synthesized as potential anticancer agents in our previous work [7]. Among of them, compound **WLJ18** (Figure 1) displayed excellent antitumor activities against various human cancer cell lines and could inactivate invasion and metastasis. These encouraging results prompted us to further design and synthesize a new class of bromophenol derivatives as potential anticancer agents.

N-containing heterocyclic compounds are important compounds with excellent biological activities, such as antiviral, anti-inflammatory and antitumor activities [8]. Especially in new drug discovery, the N-containing heterocyclic moieties were widely introduced into pharmaceutical molecules. For example, the rings of piperidine, morpholine and piperazine were introduced into anticancer drugs (Gefitinib, Vandetanib, SU11274, Sunitinib, etc. (Figure 1)) and had positive effects on activities of drugs along with several properties of molecules, including drug-target interactions, metabolic stability and toxicity [9].

Usually, the combination of different biological units leads to synergistic activity, which is an efficient strategy for the design of novel antitumor agents [10,11,12,13]. Based on the above, we designed and synthesized a series novel of bromophenol hybrids with heterocyclic molecules containing nitrogen in our continuing research for novel anticancer agents. The antitumor activities of bromophenol hybrids were screened against a series of cancer cell lines in vitro using MTT assay, and the structure-activity relationships (SARs) of these analogs are also discussed. The mechanism studies of the promising candidate compound **17a** were further investigated.

## 2. Results and Discussion

### 2.1. Chemistry

**Scheme 1:**
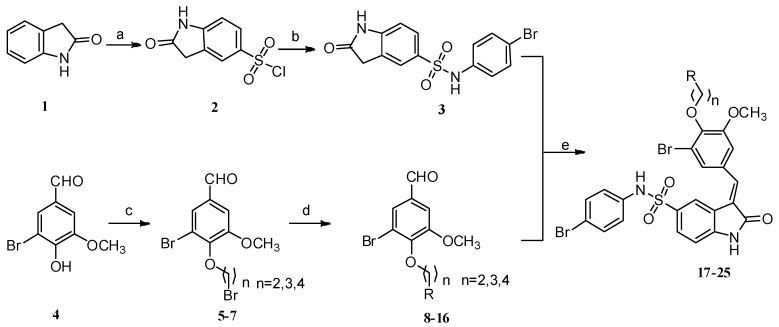
Reagents and conditions: (**a**) ClSO_3_H, 65 °C, 1 h; (**b**) 4-bromoaniline, THF, reflux; (**c**) K_2_CO_3_, DMF, 60–80 °C; (**d**) Et_3_N, DMF, 40–60 °C; and (**e**) EtOH, piperidine, reflux.

The general synthetic methods of compounds are shown in Scheme 1. Firstly, oxindole (**1**) was reacted with ClSO_3_H to yield compound **2**. Then, compound **2** and 4-bromoaniline were heated for 3 h in THF at 80 °C to afford *N*-(4-bromophenyl)-2-oxoindoline-5-sulfonamide (**3**) [7]. The aldehyde **4** was reacted with alkyl dibromide under K_2_CO_3_ in dimethylformamide (DMF) to yield intermediates **5**–**7 [14]**. Then compounds **5**–**7** were treated with the appropriate amine under Et_3_N in DMF to give compounds **8**–**16** [15]. Finally, the reactions between intermediates (**8**–**16**) and compound **3**wereperformed under the condition of Knoevenagel condensation in ethanol with a catalytic amount of piperidine to give the desired derivatives **17**–**25** in good yields. The structures of compounds **8**–**25** are shown in Table 1. All of the synthesized derivatives were purified and their structures were characterized by spectroscopic means (^1^H, ^13^C-nuclear magnetic resonance (NMR), and high-resolution mass spectrometer (HRMS)). The configuration of the double bond in compounds **7**–**25** was assigned to E based on the spectra of ^1^H NMR [16,17]. Physicochemical properties (including calculated logarithm of partition coefficient between n-octanol and H_2_O (cLogP), H_2_O solubility in mol/L (cLogS), polar surface area (TPSA), hydrogen bond acceptor (Ha), and hydrogen bond donor (Hd)), toxicity profiles (including mutagenic effect, tumorigenic effect, irritating effect and reproductive effect) and drug-likeness scores of these compounds were calculated and predicted using OSIRIS Property Explorer software at URL http://www.organic-chemistry.org/prog/peo/ [18]. The calculation of physicochemical properties and prediction of toxicity risks are shown in Table 2.

### 2.2. Biological Activity

#### 2.2.1. Cytotoxicity

All of the target compounds (**17**–**25**) were investigated for their in vitro anti-cancer activity against five human cancer cell lines, A549 (human lung cancer cell line), HepG2 (human hepatocellular carcinoma cell line), Bel7402 (human hepatocellular carcinoma cell line), HCT116 (human colorectal cancer cell line), and Caco2 (Human colonic epithelial cell line), using MTT method with sunitinib as a positive control. The IC_50_ values of these compounds are listed in Table 3.

As shown in Table 3, the rings of piperidine were introduced to bromophenol derivatives **17a** and **17b**, which exhibited excellent anticancer activities against the test cancer cell lines. Compounds **18a** and **18b** with ring of morpholine showed better activities against HCT116, Caco2 and A549 cell lines than that of compound **WLJ18**. On the contrary, the activity of compound **18c** displayed weak activities against Bel7402, HepG2, HCT116 and Caco2, except A549 with the IC_50_ value of 4.49 ± 0.73 μg/mL. When 1,4′-bipiperidine unit was introduced to bromophenol, the activities of compound **19a** and **19b** were increased. The introduction of *N*,*N*-diethylpiperidine moiety with two and three carbon chains (**20a** and **20b**) could increase the activities against the test cancer cell lines with excellent IC_50_ values. The anticancer activity of compound **21a** containing ring of 4-methylpiperazine with two-carbon chain slightly increased comparing to compound **WLJ18**. When the number of the carbon chain was two atoms, compound **21b** exhibited excellent anticancer activities, inhibiting the five cancer cell lines with IC_50_ values of 5.20 ± 0.76 μg/mL, 3.25 ± 0.32 μg/mL, 5.83 ± 1.11 μg/mL, 4.43 ± 0.53 μg/mL and 7.52 ± 0.99 μg/mL, respectively. Compound **22a** with 4-(pyrimidin-2-yl)piperazin-1-yl ring and two carbon chain showed potent activities against cancer cell lines of HCT116 and Caco2 with the IC_50_ values of 3.59 ± 0.25 μg/mL and4.09 ± 0.76 μg/mL. However, its analogs (**22b**) with three carbon chains showed weak anticancer activities. The chain length could affect the anticancer activities of these hybrids based on the above results. In an effort to gain more potent bromophenol hybrids and their information of the SARs, we probed additional structural changes. The 4-(pyrazin-2-yl) piperazin-1-yl, 4-(2-(dimethylamino)ethyl) piperazin-1-yl and bis (2-hydroxyethyl) amino groups were incorporated with **WLJ18**. Disappointingly, the anticancer activities of compounds **24** and **25** obviously decreased, which indicated that the moieties of incorporation could affect the steric clash, electron density, or hydrogen-bonding capacity, resulting different anticancer activities of bromophenol hybrids.

#### 2.2.2. Compound **17a** Induce Morphological Changes in A549 Cells

**Figure 2:**
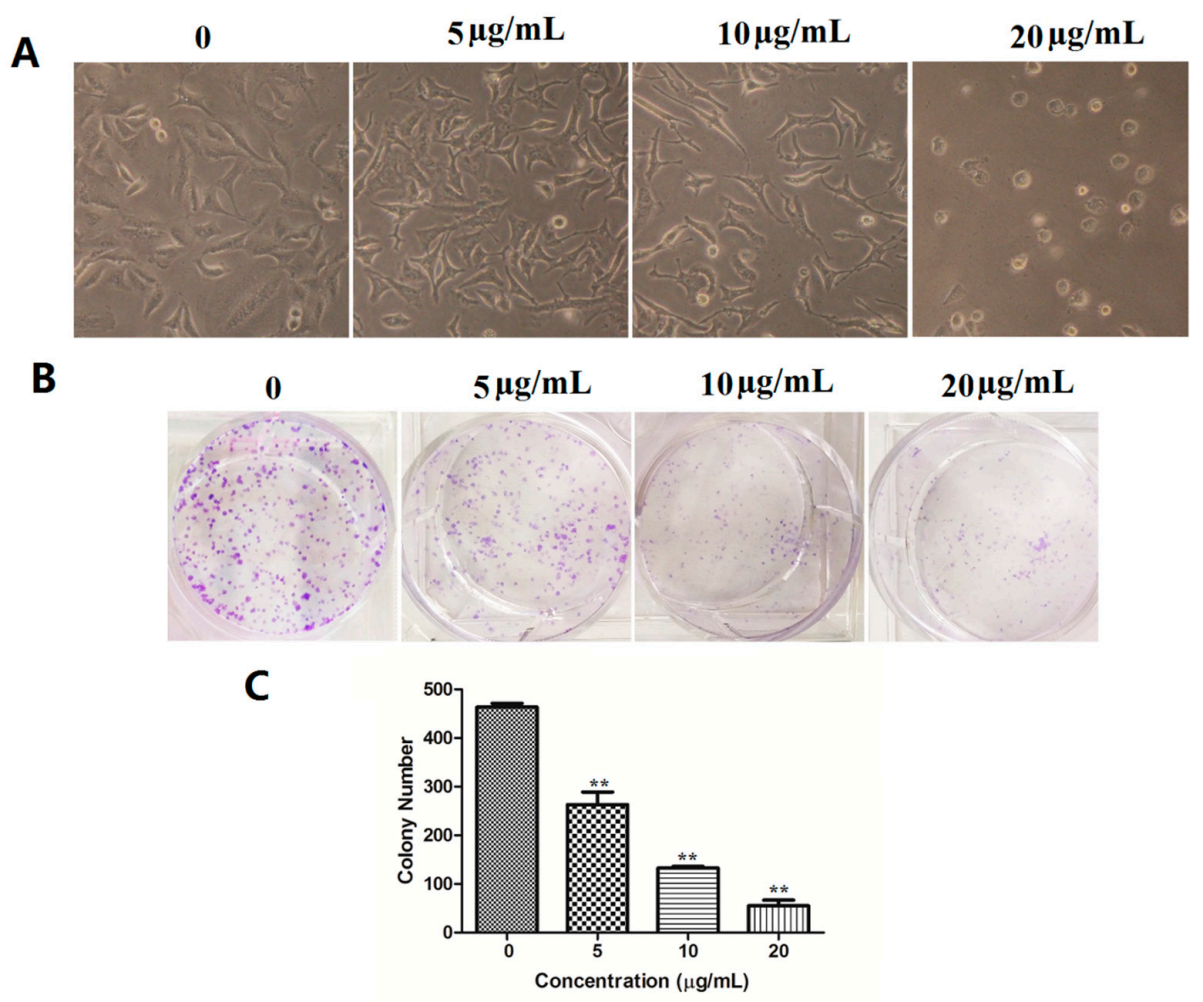
Effects of compound **17a** on cell morphology and colony formation in A549 cell. (**A**) A549 cells were treated with compound **17a** for 48 h; the representative fields were photographed at 100× magnification. (**B**,**C**) A549 cells were treated with compound **17a** (0, 5, 10, 20 µg/mL) for 10 days and colony formation was determined by staining with crystal violet. The data represent mean values (±SD) obtained from three separate experiments. ** *p* < 0.01 vs. control group.

Morphological changes of cancer cells are always associated with the growth inhibition induced by cytotoxic agents. We also took photos for the cells after treating compound **17a** for 48 h. As shown in Figure 2A, compound **17a** treated A549 cells showed morphological changes such as cell shrinkage, deformation and reduced number of viable cells.

#### 2.2.3. Compound **17a** Inhibits Colony Formation Ability of A549 Cells

The colony formation experiment was performed to determine the long-term impact of compound **17a** on A549 cells growth. A 10-day colony formation assay was performed in this study. A549 cells were seeded in six-well plates (500 cells/well). Cells were treated with various concentrations of compound **17a** (0, 5, 10, 20 µg/mL), and incubated for 10 days to allow colony formation. The results revealed that the colony-forming ability of A549 cells was significantly and dose-dependently suppressed after compound **17a** treatment (Figure 2B,C). As shown in Figure 2C, 464.5 ± 10 of colonies were present in the control panel, whereas after treatment with 5 µg/mL compound **17a** the number decreased to 263 ± 37. Further decrease to 133 ± 5 and 55 ± 17 occurred after treatment for 10 and 20 µg/mL, respectively. The results indicated that compound **17a** had a significant inhibitory effect on the colony formation of A549 cells.

#### 2.2.4. Compound **17a** Induce Apoptosis in A549 Cells

**Figure 3:**
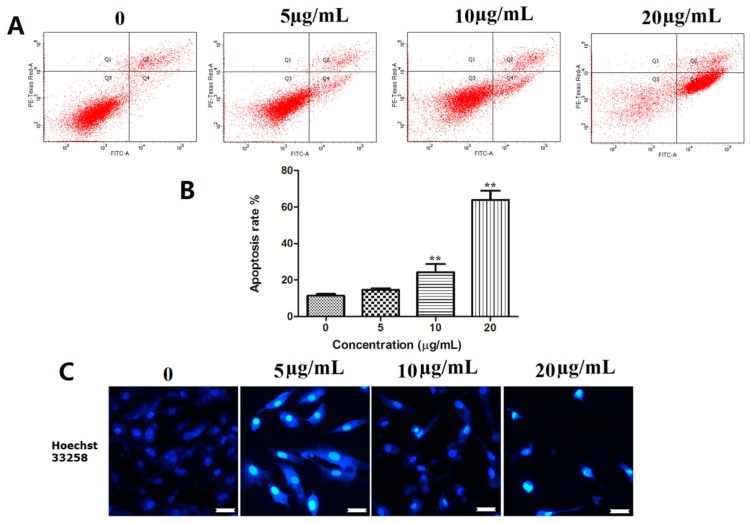
Compound **17a** induces intrinsic apoptosis in A549 cells. (**A**,**B**) Flow cytometric analysis of cells stained with Annexin V-FITC and PI. A549 cells were treated with various concentrations of compound **17a** (0, 5, 10, and 20 µg/mL) for 48 h. Cells were harvested and processed by annexin V-FITC and PI staining followed by flow cytometry analysis. (**C**) A549 cells were treated with compound **17a** (0, 5, 10, and 20 µg/mL) for 48 h. Hoechst 33258 staining was used to detected the apoptosis and photographed using an fluorescence microscopy (bar = 50 µm). ** *p* < 0.01 vs. control group.

To determine whether the compound **17a**-induced reduction in cell viability was responsible for the induction of apoptosis, A549 cells were co-stained with PI and Annexin-V FITC, and the number of apoptotic cells was estimated by flow cytometry. The flow cytometric detection of phosphatidylserine (PS) expression in early apoptosis was employed (using fluorescence-conjugated annexin-V). This combination allows the differentiation among viable cells (AV−/PI−), early-phase apoptotic cells (AV+/PI−), late-phase apoptotic cells (AV+/PI+), and necrotic cells (AV−/PI+). A dose-dependent increase in the percentage of apoptotic cells was noted after the cells were treated for 48 h with compound **17a** (0, 5, 10, 20 µg/mL). As shown in Figure 3A,B, 11.45 ± 1.20% of apoptotic cells were present in the control panel, whereas, after treatment with 5 µg/mL compound **17a**, the population rose to 14.65 ± 1.06%. Further increase to 24.3 ± 6.36% and 63.8 ± 7.21% occurred after treatments of 10 and 20 µg/mL, respectively.

#### 2.2.5. Compound **17a** Causes DNA Fragmentations and Morphological Changes

An essential hallmark of apoptosis is DNA fragmentation and morphological changes. Morphological changes of apoptotic cells such as nuclear apoptotic bodies were analyzed by fluorescence microscopy with Hoechst 33258 staining. A549 cells were treated with 5, 10 and 20 μg/mL compound **17a** for 48 h. As shown in Figure 3C, the treatment of the A549 cells with compound **17a** resulted in the induction of chromatin condensation, fragmentation and clear apoptotic bodies that were visualized in fluorescence microscopy.

#### 2.2.6. Compound **17a** Induce G0/G1 Cell Cycle Arrest in A549 Cells

**Figure 4:**
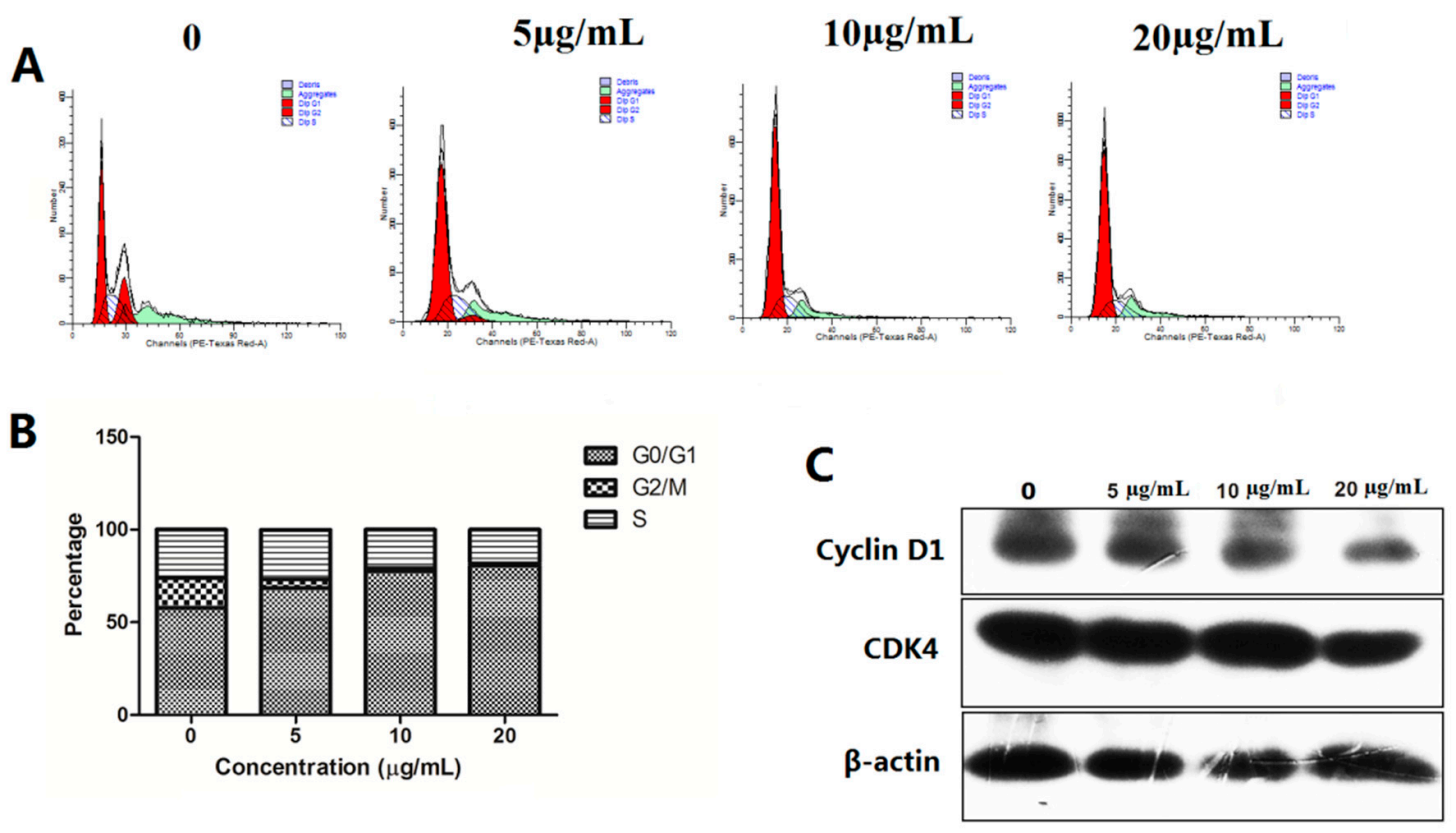
Compound **17a** induces G0/G1 cell cycle arrest in A549 cells. (**A**,**B**) A549 cells were treated with compound **17a** (0, 5, 10, and 20 µg/mL) for 48 h. Cells were harvested and fixed in 70% ethanol overnight, and then cells were stained with PI and analysis by FACS. (**C**) Western blot analysis of cell cycle-related proteins, including cyclin D1 and CDK4. β-actin was used to normalize protein content. The data represent mean values (±SD) obtained from three separate experiments.

To elucidate whether the cytotoxicity induced by the derivatives was due to cell cycle arrest, A549 cells were treated with compound **17a** (0, 5, 10, 20 µg/mL) for 48 h. Flow cytometry analysis showed A549 cells, which were treated with compound **17a**, arrested in G0/G1 phase in a dose-dependent manner (Figure 4). When compared with control, compound **17a** increased the population in the G1 phase from 57.85% to 80.63% at concentration of 20 µg/mL, while the G2/M phase was decreased. These findings denote that compound **17a** can induce cell cycle arrest in G0/G1 phase.

Cell cycle is regulated by a family of protein kinase complexes, including CDKs and cyclins, in eukaryotic cells [19]. The previous study has reported that the reduced activities of CDK 4 and cyclin D1 are the hallmarks of cell cycle arrest at the G1/S phase [20,21]. In this study, Western blot analysis showed that treatment of A549 cells with compound **17a** at 20 µg/mL significantly decreased the level of cyclin D1 and CDK4.

#### 2.2.7. Compound **17a** Triggers ROS Generation

**Figure 5:**
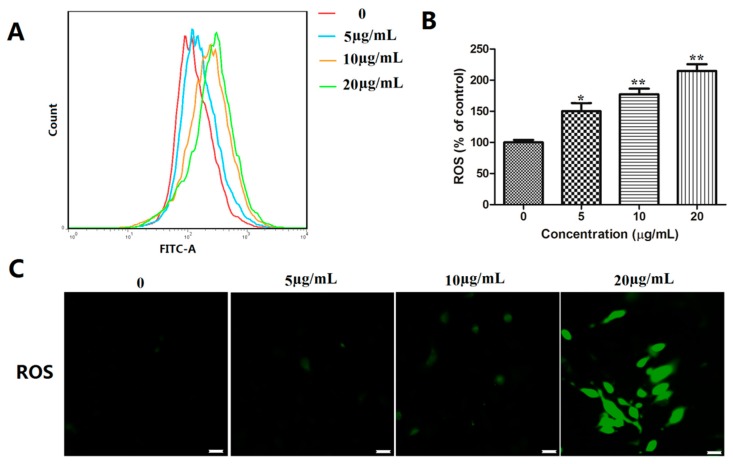
Compound **17a** induces ROS generation in A549 cells. A549 cells were treated with various concentrations of compound **17a** (0, 5, 10, and 20 µg/mL) for 48 h; the medium was discarded and cells were incubated at 37 °C in the dark for 20 min with culture medium containing DCFH-DA. (**A**,**B**) Cells were harvested and analyzed using FACS. (**C**) Cells were washed twice with PBS and analyzed using fluorescence microscopy (bar = 50 µm). All data were representative of three independent experiments. * *p* < 0.05; ** *p* < 0.01 vs. control group.

ROS are highly harmful elements to cells as they initiate oxidative stress and ultimately cause cellular damage. Excessive ROS generation renders cells vulnerable to apoptosis. To determine whether compound **17a** triggers ROS generation in A549 cells to induce apoptosis, the ROS level was measured using 2′,7′-dichlorodihydrofluoresce in diacetate (DCFH-DA) as fluorescent probe. DCFH-DA is cleaved by intracellular esterases into its non-fluorescent form (DCFH), which is converted to a green fluorescent product, carboxy-DCF, via oxidation. A rapid production of ROS occurred after the exposure of A549 cells to compound **17a** (Figure 5A,B). When compared with control (100%), the mean DCF fluorescence increased by 150.10 ± 22.60%, 177.32 ± 15.60% and 214.60 ± 18.90%, when treated with **17a** for 48 h. Treatment of compound **17a** also increased the green fluorescence intensity in A549 cells (Figure 5C).

#### 2.2.8. Effect of Compound **17a** on the Expression of Apoptosis-Related Proteins

**Figure 6:**
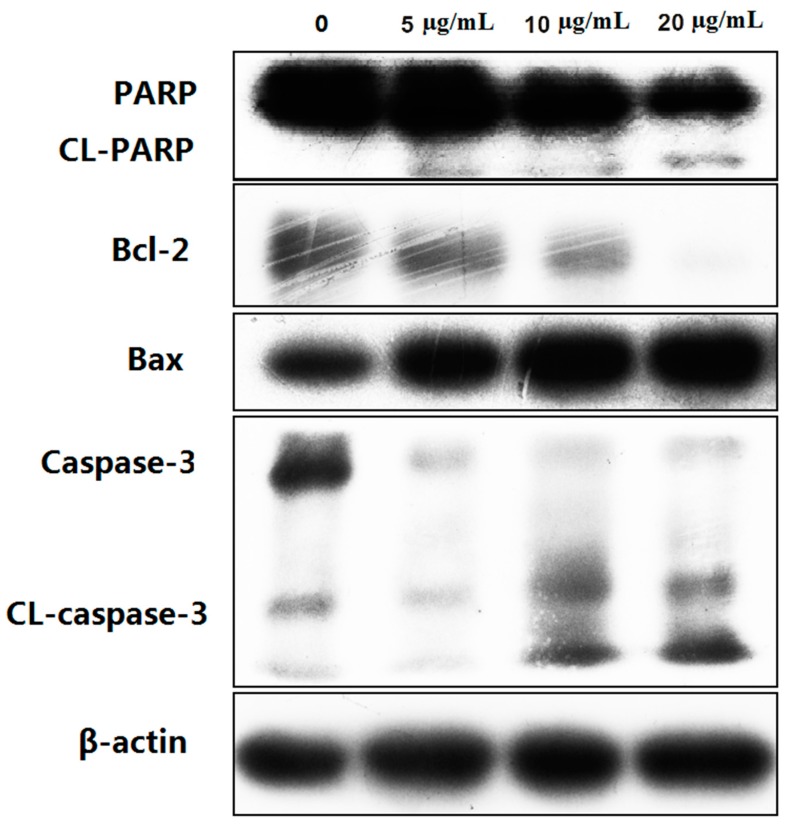
Western blot analysis of the effect of compound **17a** on apoptosis-related proteins. A549 cells were treated with various concentrations of compound **17a** (0, 5, 10, and 20 µg/mL) for 48 h. Western blot analyses were performed, and β-actin was used to normalize protein content. All data were representative of three independent experiments.

The Bcl-2 family members are important regulators of mitochondrial function during apoptosis. It has been recognized that accumulation of ROS does not kill cells directly, it triggers an apoptotic signing pathway that leads to cell death, such as increase the Bax/Bcl-2 ratio and activate caspase-3 and PARP [22]. In our study, the treatment of A549 cells induces decrease in the expression of the anti-apoptotic proteins Bcl-2 and down-regulation in the expression levels of Bcl-2 (Figure 6). Treatment with compound **17a** also activated caspase-3 and PARP in A549 cells (Figure 6). All these data demonstrated that compound **17a** induced A549 cells apoptosis in vitro, probably through the ROS-mediated apoptotic pathway.

## 3. Materials and Methods

### 3.1. Chemistry

Reaction reagents were purchased from J&K Scientific Ltd. (Beijing, China). Organic solvents were analytical reagent grade and purchased from Tianjin Chemical Reagent Co., Ltd. (Tianjin, China) Column chromatography (CC): silica gel (200–300 mesh; Qingdao Makall Group Co., Ltd.; Qingdao, China). All reactions were monitored using thin-layer chromatography (TLC) on silica gel plates. ^1^H and ^13^C-NMR spectra were recorded on Bruker DRX 500 MHz spectrometers with tetramethylsilane (TMS) as the internal standard (Bruker, Bremerhaven, Germany). MS and HRMS spectra were determined on a LCMS-IT-TOF mass spectrometer (Shimadzu, Kyoto, Japan). Melting points were determined on a SGW X-4 Melting Point Apparatus (Shanghai Precision Science Instrument Co., Ltd.; Shanghai, China). The synthesized compounds were named using ChemBioDraw Ultra software (v 12.0, PerkinElmer, Waltham, MA, USA).

#### 3.1.1. General Procedures for the Preparation of Compounds **5**–**7**

The mixture of anhydrous K_2_CO_3_ (1.00 eq.) and the 3-bromo-4-hydroxy-5-methoxybenzaldehyde 4 (1.00 eq.) were suspended in dry DMF. Then, an alkyl dibromide (1.10 eq.) was added in several portions (neat) during 0.5 h. This reaction mixture was stirred at 60–80 °C overnight. After complete turnover, H_2_O was added and the aqueous solution was extracted three times with chloroform. The combined organic layers washed with H_2_O, HCl and brine. Then, the organic layer was dried with Na_2_SO_4_ and removed in vacuo. The resulting residue was purified by column chromatography on silica gel to provide compounds **5**–**7**. Details of synthesis of compounds **5**–**7** are given in the Supplementary Data.

#### 3.1.2. General Procedure for the Preparation of Compounds **8**–**16**

To a solution of compounds **5**–**7** (0.1 mmol) in DMF, the appropriate amine (0.3 mmol) and Et_3_N (160 μL, 1.17 mmol) was added. After stirring at room temperature (unless otherwise indicated) overnight, the mixture was poured into H_2_O. Then, the organic phase was extracted three times with EtOAc (4 × 30 mL) and washed with H_2_O (2 × 30 mL) and with brine (2 × 30 mL). The solvent was evaporated, and the residue was purified by column chromatography on silica gel to provide the compounds **8**–**16**. Details of synthesis of compounds **8**–**16** are given in the Supplementary Data.

#### 3.1.3. General Procedures for the Preparation of Compounds **17**–**25**

The synthesis of compounds **2** and **3** were performed as our previous report [7]. Piperidine (50 μL) was added to a mixture of compound **3** (0.5 mmol) and appropriate aldehydes **8**–**16** (0.55 mmol) in ethanol (5 mL). The reaction mixture was heated to refluxing and stirred for 2 h, and TLC analysis indicated when the reaction was complete. The crude product was filtered, washed with ethanol and dried in a vacuum (if no solid precipitated, the crude product was chromatographed using a silica gel column) to afford the title compounds **17**–**25** as yellow solid.

*(E)-3-(3-Bromo-5-methoxy-4-(2-(piperidin-1-yl)ethoxy)benzylidene)-N-(4-bromophenyl)-2-oxoindoline-5-sulfonamide* (**17a**). Intermediate **8a** was treated with compound 3 following the general procedure to give the desired product **17a**. Yield: 56.4%; mp.: 210–215 °C; ^1^H-NMR (DMSO-*d*_6_, 500 MHz, ppm): δ 11.05 s, 1H), 7.58–7.63 overlap, 2H), 7.49 (s, 1H), 7.42 (s, 1H), 7.39 (d, 1H, *J =* 9.0 Hz), 7.31 (d, 2H, *J =* 8.5 Hz), 7.05 (d, 1H, *J =* 9.0 Hz), 6.94 (d, 2H, *J =* 8.5 Hz), 4.13 (t, 2H, *J =* 6.0 Hz), 3.87 (s, 3H), 2.68 (t, 2H, *J =* 6.0 Hz), 2.41 (m, 4H), 1.43 (m, 4H) 1.32 (m, 2H); ^13^C-NMR (DMSO-*d*_6_, 125 MHz, ppm): δ 168.9, 152.6, 147.7, 146.9, 138.4, 138.2, 137.3, 132.4(2C), 131.1, 129.6, 126.6, 125.6, 122.3(2C), 121.4, 117.8, 116.8, 113.3, 110.7, 110.2, 71.0, 58.5, 56.6, 54.7(2C), 25.9(2C), 24.4; ESIMS: *m/z* 690 [M + H]^+^ HRESIMS: calc. for C_29_H_29_Br_2_N_3_O_5_S [M + H]^+^ 690.0289, found 690.0267.

*(E)-3-(3-Bromo-5-methoxy-4-(3-(piperidin-1-yl)propoxy)benzylidene)-N-(4-bromophenyl)-2-oxoindoline-5-sulfonamide* (**17b**). Intermediate **8b** was treated with compound 3 following the general procedure to give the desired product **17b**. Yield: 61.6%; mp.: 198–202 °C; ^1^H-NMR (DMSO-*d*_6_, 500 MHz, ppm): δ 11.08 (s, 1H), 7.59–7.63 (overlap, 2H), 7.49 (s, 1H), 7.42 (s, 1H), 7.38 (d, 1H, *J =* 9.0 Hz), 7.30 (d, 2H, *J =* 8.5 Hz), 7.05 (d, 1H, *J =* 9.0 ), 6.94 (d, 2H, *J =* 8.5 Hz), 4.07 (t, 2H, *J =* 6.0 Hz), 3.87 (s, 3H), 2.43 (t, 2H, *J =* 6.0 Hz), 2.34 (m, 4H), 1.88 (m, 2H), 1.45 (m, 4H), 1.34 (m, 2H); ^13^C-NMR (DMSO-*d*_6_, 125 MHz, ppm): δ 168.9, 152.9, 147.6, 146.8, 138.4, 138.2, 137.2, 132.4(2C), 131.1, 129.6, 126.5, 125.4, 122.3(2C), 121.3, 117.8, 116.8, 113.4, 110.8, 110.2, 72.1, 56.6, 55.5, 54.4(2C), 27.6, 26.0(2C), 24.5; ESIMS: *m/z* 704 [M + H]^+^ HRESIMS: calc. for C_30_H_31_N_3_O_5_SBr_2_ [M + H]^+^ 704.0424, found 704.0466.

*(E)-3-(3-Bromo-5-methoxy-4-(2-morpholinoethoxy)benzylidene)-N-(4-bromophenyl)-2-oxoindoline-5-sulfonamide* (**18a**). Intermediate **9a** was treated with compound **3** following the general procedure to give the desired product **18a**. Yield: 65.2%; mp.: 238–242 °C; ^1^H-NMR (DMSO-*d*_6_, 500 MHz, ppm): δ 11.08 (s, 1H), 7.58–7.64 (overlap, 2H), 7.50 (s, 1H), 7.43 (s, 1H), 7.40 (d, 1H, *J =* 9.0 Hz), 7.32 (d, 2H, *J =* 8.5 Hz), 7.05 (d, 1H, *J =* 9.0 Hz), 6.95 (d, 2H, *J =* 8.5 Hz), 4.12 (t, 2H, *J =* 6.0 Hz), 3.87 (s, 3H), 3.51 (m, 4H), 2.67 (t, 2H, *J =* 6.0 Hz), 2.46 (m, 4H); ^13^C-NMR (DMSO-*d*_6_, 125 MHz, ppm): δ 168.9, 153.6, 147.6, 146.8, 144.5, 137.8, 132.4(2C), 132.1, 131.1, 129.6, 126.6, 125.6, 122.3(2C), 121.3, 119.1, 117.8, 116.8, 113.2, 110.9, 70.6, 66.6(2C), 58.2, 56.6, 53.9(2C); ESIMS: *m/z* 692 [M + H]^+^ HRESIMS: calc. for C_28_H_27_N_3_O_6_SBr_2_ [M + H]^+^ 692.0060, found 692.0072.

*(E)-3-(3-Bromo-5-methoxy-4-(3-morpholinopropoxy)benzylidene)-N-(4-bromophenyl)-2-oxoindoline-5-sulfonamide* (**18b**). Intermediate **9b** was treated with compound **3** following the general procedure to give the desired product **18b**. Yield: 55.3%; mp.: 125–130 °C; ^1^H-NMR (DMSO-*d*_6_, 500 MHz, ppm): δ 11.08 (s, 1H), 7.58–7.64 (overlap, 2H), 7.49 (s, 1H), 7.39–7.42 (overlap, 2H), 7.31 (d, 2H, *J =* 8.5 Hz), 7.05 (d, 1H, *J =* 9.0 Hz), 6.94 (d, 2H, *J =* 8.5 Hz), 4.09 (t, 2H, *J =* 6.0 Hz), 3.86 (s, 3H), 3.55 (m, 4H), 2.48 (m, 2H), 2.36 (m, 4H)), 1.89 (m, 2H); ^13^C-NMR (DMSO-*d*_6_, 125 MHz, ppm):δ 168.7, 153.7, 147.6, 146.8, 144.5, 137.8, 132.4(2C), 132.0, 131.1, 129.6, 126.6, 125.5, 122.3(2C), 121.3, 119.1, 117.8, 116.9, 113.4, 110.8, 71.9, 66.7(2C), 56.6, 55.2, 53.8(2C), 27.3; ESIMS: *m/z* 706 [M + H]^+^ HRESIMS: calc. for C_29_H_29_N_3_O_6_SBr_2_ [M + H]^+^ 706.0217, found 706.0248.

*(E)-3-(3-Bromo-5-methoxy-4-(4-morpholinobutoxy)benzylidene)-N-(4-bromophenyl)-2-oxoindoline-5-sulfonamide* (**18c**). Intermediate **9c** was treated with compound **3** following the general procedure to give the desired product **18c**. Yield: 67.7%; mp.: 170–175 °C; ^1^H-NMR (DMSO-*d*_6_, 500 MHz, ppm): δ 11.10 (s, 1H), 7.59–7.64 (overlap, 2H), 7.49 (s, 1H), 7.38–7.42 (overlap, 2H), 7.31 (d, 2H, *J =* 8.5 Hz), 7.06 (d, 1H, *J =* 9.0 Hz), 6.94 (d, 2H, *J =* 8.5 Hz), 4.06 (t, 2H, *J =* 6.0 Hz), 3.86 (s, 3H), 3.53 (m, 4H), 2.26–2.32 (overlap, 6H), 1.74 (m, 2H), 1.60 (m, 2H); ^13^C-NMR (DMSO-*d*_6_, 125 MHz, ppm):δ 168.9, 153.7, 147.6, 146.8, 144.4, 138.0, 132.4(2C), 132.4, 131.2, 129.6, 126.6, 125.5, 122.3(2C), 121.3, 119.1, 117.8, 116.9, 113.4, 110.8, 73.4, 66.7(2C), 58.3, 56.6, 53.8(2C), 28.1, 22.9; ESIMS: *m/z* 720 [M + H]^+^ HRESIMS: calc. for C_30_H_31_N_3_O_6_SBr_2_ [M + H]^+^ 720.0373, found 720.0396.

*(E)-3-(4-(2-([1,4′-Bipiperidin]-1*′*-yl)ethoxy)-3-bromo-5-methoxybenzylidene)-N-(4-bromophenyl)-2-oxoindoline-5-sulfonamide* (**19a**). Intermediate **10a** was treated with compound **3** following the general procedure to give the desired product **19a**. Yield:59.1%; mp.: 202–207 °C; ^1^H-NMR (DMSO-*d*_6_, 500 MHz, ppm): δ 11.08 (s, 1H), 7.65–7.67 (overlap, 3H), 7.39 (d, 1H, *J =* 9.0 Hz), 7.38 (s, 1H), 7.27 (d, 2H, *J =* 8.5 Hz), 6.99 (d, 1H, *J =* 9.0 Hz), 6.93 (d, 2H, *J =* 8.5 Hz), 4.22 (t, 2H, *J =* 6.0 Hz), 3.92 (s, 3H), 2.90 (t, 2H, *J =* 6.0 Hz), 2.44–2.48 (overlap, 9H), 1.54 (m, 8H), 1.30 (m, 2H); ^13^C-NMR (DMSO-*d*_6_, 125 MHz, ppm): δ 169.7, 153.5, 146.9, 146.2, 137.4, 136.8, 132.3, 131.8(2C), 130.9, 129.0, 126.4, 126.2, 122.6(2C), 121.4, 117.7, 117.3, 112.2, 110.9, 110.2, 69.9, 63.4, 56.9, 55.7, 51.9(2C), 49.9(2C), 29.6(2C), 26.0(2C), 23.5; ESIMS: *m/z* 773 [M + H]^+^ HRESIMS: calc. for C_34_H_38_N_4_O_5_SBr_2_ [M + H]^+^ 773.1002, found 773.1114.

*(E)-3-(4-(3-([1,4′-Bipiperidin]-1*′*-yl)propoxy)-3-bromo-5-methoxybenzylidene)-N-(4-bromophenyl)-2-oxoindoline-5-sulfonamide* (**19b**). Intermediate **10b** was treated with compound **3** following the general procedure to give the desired product **19b**. Yield: 67.5%; mp.: 160–165 °C; 1H-NMR (DMSO-*d*_6_, 500 MHz, ppm): δ 11.11 (s, 1H), 7.65–7.67 (overlap, 3H), 7.43 (s, 1H), 7.39 (d, 1H, *J =* 9.0 Hz), 7.30 (d, 2H, *J =* 8.5 Hz), 7.07 (d, 1H, *J =* 9.0 Hz), 7.00 (d, 2H, *J =* 8.5 Hz), 4.20 (t, 2H, *J =* 6.0 Hz), 3.88 (s, 3H), 2.99 (t, 2H, *J =* 6.0 Hz), 2.48–2.53 (overlap, 6H), 2.25 (m, 1H), 1.89–1.97 (overlap, 4H), 1.75 (m, 2H), 1.55 (m, 6H), 1.38 (m, 2H); ^13^C-NMR (DMSO-*d*_6_, 125 MHz, ppm): δ 168.9, 153.7, 146.9, 146.7, 137.7, 137.2, 132.4, 132.3(2C), 131.2, 129.6, 126.4, 125.4, 122.3(2C), 121.3, 117.7, 116.9, 113.4, 110.8, 110.2, 71.9, 62.5, 56.6, 54.4, 52.5(2C), 49.7(2C), 27.6, 26.7(2C), 24.6(2C), 23.4; ESIMS: *m/z* 787 [M + H]^+^ HRESIMS: calc. for C_35_H_40_N_4_O_5_SBr_2_ [M + H]^+^ 787.1159, found 787.1232.

*(E)-3-(3-Bromo-4-(2-(4-(diethylamino)piperidin-1-yl)ethoxy)-5-methoxybenzylidene)-N-(4-bromophenyl)-2-oxoindoline-5-sulfonamide* (**20a**). Intermediate **11a** was treated with compound 3 following the general procedure to give the desired product **20a**. Yield: 64.2%; mp.: 148–152 °C; ^1^H-NMR (DMSO-*d*_6_, 500 MHz, ppm): δ 11.10 (s, 1H), 7.66 (s, 1H), 7.64 (s, 1H), 7.58 (s, 1H), 7.28 (d, 1H, *J =* 9.0 Hz), 7.24 (s, 1H), 7.20 (d, 2H, *J =* 8.5 Hz), 7.03 (d, 1H, *J =* 9.0 Hz), 6.90 (d, 2H, *J =* 8.5 Hz), 4.21 (t, 2H, *J =* 6.0 Hz), 3.89 (s, 3H), 3.16 (d, 2H, *J =* 11.5 Hz), 2.82 (t, 2H, *J =* 6.0 Hz), 2.62–2.69 (overlap, 5H), 2.10 (t, 2H, *J =* 6.0 Hz), 1.80 (d, 2H, *J =* 11.5 Hz), 1.57 (m, 2H), 1.06 (t, 6H, *J =* 7.0 Hz); ^13^C-NMR (DMSO-*d*_6_, 125 MHz, ppm): δ 169.7, 153.5, 146.8, 146.1, 138.0, 137.4, 133.0, 131.7(2C), 130.9, 129.1, 126.3, 125.5, 122.6(2C), 121.4, 117.7, 116.5, 112.2, 110.9, 110.1, 70.1, 57.8, 57.4, 55.6, 53.0(2C), 43.3(2C), 27.0(2C), 11.2(2C); ESIMS: *m/z* 761 [M + H]^+^ HRESIMS: calc. for C_33_H_38_N_4_O_5_SBr_2_ [M + H]^+^ 761.1002, found 761.1140.

*(E)-3-(3-Bromo-4-(3-(4-(diethylamino)piperidin-1-yl)propoxy)-5-methoxybenzylidene)-N-(4-bromophenyl)-2-oxoindoline-5-sulfonamide* (**20b**). Intermediate **11b** was treated with compound **3** following the general procedure to give the desired product **20b**. Yield: 61.3%; mp.: 135–142 °C; ^1^H-NMR (DMSO-*d*_6_, 500 MHz, ppm): δ 11.12 (s, 1H), 7.63–7.60 (overlap, 3H), 7.42 (s, 1H), 7.38 (d, 1H, *J =* 9.0 Hz), 7.29 (d, 2H, *J =* 8.5 Hz), 7.05 (d, 1H, *J =* 9.0 Hz), 6.96 (d, 2H, *J =* 8.5 Hz), 4.07 (t, 2H, *J =* 6.0 Hz), 3.87 (s, 3H), 2.94 (d, 2H, *J =* 11.0 Hz), 2.48–2.60 (overlap, 7H), 1.82–1.98 (m, 4H), 1.73 (d, 2H, *J =* 11.0 Hz), 1.50 (m, 2H), 1.00 (t, 6H, *J =* 7.0 Hz); ^13^C-NMR (DMSO-*d*_6_, 125 MHz, ppm): δ 168.9, 153.5, 147.0, 146.8, 144.4, 138.0, 137.2, 132.2(2C), 131.1, 129.6, 126.6, 125.5, 122.3(2C), 121.3, 117.7, 116.7, 113.4, 110.8, 110.2, 71.9, 58.4, 56.6, 54.6, 52.8(2C), 43.6(2C), 27.7(2C), 26.8, 12.6(2C); ESIMS: *m/z* 773 [M + H]^+^ HRESIMS: calc. for C_34_H_40_N_4_O_5_SBr_2_ [M + H]^+^ 773.1013, found 773.0970.

*(E)-3-(3-Bromo-5-methoxy-4-(2-(4-methylpiperazin-1-yl)ethoxy)benzylidene)-N-(4-bromophenyl)-2-oxoindoline-5-sulfonamide* (**21a**). Intermediate **12a** was treated with compound **3** following the general procedure to give the desired product **21a**. Yield: 58.6%; mp.: 123–127 °C; ^1^H-NMR (DMSO-*d*_6_, 500 MHz, ppm): δ 11.08 (s, 1H), 7.64 (s, 1H), 7.60 (s, 1H), 7.49 (s, 1H), 7.42 (s, 1H), 7.39 (d, 1H, *J =* 9.0 Hz), 7.30 (d, 2H, *J =* 8.5 Hz), 7.06 (d, 1H, *J =* 9.0 Hz), 6.95 (d, 2H, *J =* 8.5 Hz), 4.12 (t, 2H, *J =* 6.0 Hz), 3.86 (s, 3H), 2.71 (t, 2H, *J =* 6.0 Hz), 2.48 (m, 4H), 2.33 (m, 4H), 2.11 (s, 3H); ^13^C-NMR (DMSO-*d*_6_, 125 MHz, ppm): δ 168.8, 153.6, 147.7, 146.8, 144.4, 138.0, 132.3(2C), 132.0, 131.2, 129.6, 126.6, 125.6, 122.3(2C), 121.3, 119.1, 117.8, 116.8, 113.3, 110.8, 70.0, 57.8(2C), 56.5, 55.1(2C), 53.2, 46.1; ESIMS: *m/z* 705 [M + H]^+^ HRESIMS: calc. for C_29_H_30_N_4_O_5_SBr_2_ [M + H]^+^ 705.0376, found 705.0399.

*(E)-3-(3-Bromo-5-methoxy-4-(3-(4-methylpiperazin-1-yl)propoxy)benzylidene)-N-(4-bromophenyl)-2-oxoindoline-5-sulfonamide* (**21b**). Intermediate **12b** was treated with compound **3** following the general procedure to give the desired product **21b**. Yield: 64.2%; mp.: 120–125 °C; ^1^H-NMR (DMSO-*d*_6_, 500 MHz, ppm): δ 11.08 (s, 1H), 7.63 (s, 1H), 7.60 (s, 1H), 7.49 (s, 1H), 7.42 (s, 1H), 7.39 (d, 1H, *J =* 9.0 Hz), 7.30 (d, 2H, *J =* 8.5 Hz), 7.05 (d, 1H, *J =* 9.0 Hz), 6.95 (d, 2H, *J =* 8.5 Hz), 4.09 (t, 2H, *J =* 6.0 Hz), 3.86 (s, 3H), 2.23–2.50 (overlap, 10H), 2.12 (s, 3H), 1.83 (m, 2H); ^13^C-NMR (DMSO-*d*_6_, 125 MHz, ppm): δ 168.9, 153.6, 147.6, 146.8, 144.5, 137.9, 132.4(2C), 132.2, 131.2, 129.6, 126.4, 125.5, 122.2(2C), 121.3, 119.1, 117.8, 116.8, 113.4, 110.8, 71.9, 56.6, 55.2(2C), 54.7, 53.1(2C), 46.1, 27.6; ESIMS: *m/z* 719 [M + H]^+^ HRESIMS: calc. for C_30_H_32_N_4_O_5_SBr_2_ [M + H]^+^ 719.0533, found 719.0579.

*(E)-3-(3-Bromo-5-methoxy-4-(4-(4-methylpiperazin-1-yl)butoxy)benzylidene)-N-(4-bromophenyl)-2-oxoindoline-5-sulfonamide* (**21c**). Intermediate **12c** was treated with compound **3** following the general procedure to give the desired product **21c**. Yield: 58.7%; mp.: 135–140 °C; ^1^H-NMR (DMSO-*d*_6_, 500 MHz, ppm): δ 11.09 (s, 1H), 7.82 (s, 1H), 7.60 (s, 1H), 7.48 (s, 1H), 7.42 (s, 1H), 7.39 (d, 1H, *J =* 9.0 Hz), 7.31 (d, 2H, *J =* 8.5 Hz), 7.06 (d, 1H, *J =* 9.0 Hz), 6.96 (d, 2H, *J =* 8.5 Hz), 4.05 (t, 2H, *J =* 6.0 Hz), 3.86 (s, 3H), 2.33–2.48 (overlap, 10H), 2.21 (s, 3H), 1.59–1.79 (m, 4H); ^13^C-NMR (DMSO-*d*_6_, 125 MHz, ppm): δ 169.2, 153.6, 148.4, 146.4, 143.5, 139.6, 137.8, 132.4(2C), 131.9, 128.2, 126.4, 125.5, 122.2(2C), 120.8, 118.0, 116.5, 116.2, 115.6, 110.5, 73.5, 57.5, 56.3, 54.7(2C), 52.5(2C), 45.6, 28.0, 26.7; ESIMS: *m/z* 733 [M + H]^+^ HRESIMS: calc. for C_31_H_34_N_4_O_5_SBr_2_ [M + H]^+^ 733.0689, found 733.0711.

*(E)-3-(3-Bromo-5-methoxy-4-(2-(4-(pyrimidin-2-yl)piperazin-1-yl)ethoxy)benzylidene)-N-(4-bromophenyl)-2-oxoindoline-5-sulfonamide* (**22a**). Intermediate **13a** was treated with compound **3** following the general procedure to give the desired product **22a**. Yield: 61.2%; mp.: 184–190 °C; ^1^H-NMR (DMSO-*d*_6_, 500 MHz, ppm): δ 11.08 (s, 1H), 8.31 (d, 2H, *J =* 4.5 Hz), 7.64 (s, 1H), 7.60 (s, 1H), 7.50 (s, 1H), 7.43 (s, 1H), 7.39 (d, 1H, *J =* 9.0 Hz), 7.31 (d, 2H, *J =* 8.5 Hz), 7.06 (d, 1H, *J =* 9.0 Hz), 6.96 (d, 2H, *J =* 8.5 Hz), 6.58 (t, 1H, *J =* 4.5 Hz), 4.18 (t, 2H, *J =* 6.0 Hz), 3.87 (s, 3H), 3.66 (t, 4H, *J =* 5.5 Hz), 2.73 (t, 2H, *J =* 5.5 Hz), 2.50 (t, 4H, *J =* 4.5 Hz); ^13^C-NMR (DMSO-*d*_6_, 125 MHz, ppm): δ 168.9, 161.6, 158.3(2C), 153.6, 147.6, 146.7, 144.5, 137.6, 132.4(2C), 132.1, 131.2, 129.6, 126.6, 125.6, 122.3(2C), 121.4, 119.1, 117.8, 116.8, 113.4, 110.5, 110.2, 70.9, 57.8, 56.6, 53.1(2C), 43.8(2C); ESIMS: *m/z* 769 [M + H]^+^ HRESIMS: calc. for C_32_H_30_N_6_O_5_SBr_2_ [M + H]^+^ 769.0438, found 769.0447.

*(E)-3-(3-Bromo-5-methoxy-4-(3-(4-(pyrimidin-2-yl)piperazin-1-yl)propoxy)benzylidene)-N-(4-bromophenyl)-2-oxoindoline-5-sulfonamide* (**22b**). Intermediate **13b** was treated with compound **3** following the general procedure to give the desired product **22b**. Yield: 66.5%; mp.: 123–127 °C; ^1^H-NMR (DMSO-*d*_6_, 500 MHz, ppm): δ 11.06 (s, 1H), 8.32 (d, 2H, *J =* 4.5 Hz), 7.64 (s, 1H), 7.60 (s, 1H), 7.50 (s, 1H), 7.43 (s, 1H), 7.39 (d, 1H, *J =* 9.0 Hz), 7.31 (d, 2H, *J =* 8.5 Hz), 7.06 (d, 1H, *J =* 9.0 Hz), 6.96 (d, 2H, *J =* 8.5 Hz), 6.58 (t, 1H, *J =* 4.5 Hz), 4.13 (t, 2H, *J =* 6.0 Hz), 3.87 (s, 3H), 3.69 (t, 4H, *J =* 5.5 Hz), 2.55 (t, 2H, *J =* 5.5 Hz), 2.42 (t, 4H, *J =* 4.5 Hz), 1.93 (2H); ^13^C-NMR (DMSO-*d*_6_, 125 MHz, ppm): δ 168.9, 161.6, 158.3(2C), 153.6, 147.6, 146.7, 144.4, 137.7, 132.4(2C), 132.0, 131.2, 129.6, 126.6, 125.5, 122.3(2C), 121.3, 119.1, 117.8, 116.8, 113.4, 110.5, 110.3, 71.9, 56.6, 54.9, 53.0(2C), 43.8(2C), 27.6; ESIMS: *m/z* 783 [M + H]^+^ HRESIMS: calc. for C_33_H_32_N_6_O_5_SBr_2_ [M + H]^+^ 783.0594 found 783.0601.

*(E)-3-(3-Bromo-5-methoxy-4-(2-(4-(pyrazin-2-yl)piperazin-1-yl)ethoxy)benzylidene)-N-(4-bromophenyl)-2-oxoindoline-5-sulfonamide* (**23a**). Intermediate **14a** was treated with compound **3** following the general procedure to give the desired product **23a**. Yield: 56.2%; mp.: 143–151 °C; ^1^H-NMR (DMSO-*d*_6_, 500 MHz, ppm): δ 11.07 (s, 1H), 8.27 (s, 1H), 8.03 (s, 1H), 7.79 (s, 1H), 7.64 (s, 1H), 7.60 (s, 1H), 7.50 (s, 1H), 7.43 (s, 1H), 7.38 (d, 1H, *J =* 9.0 Hz), 7.31 (d, 2H, *J =* 8.5 Hz), 7.06 (d, 1H, *J =* 9.0 Hz), 6.96 (d, 2H, *J =* 8.5 Hz), 4.18 (t, 2H, *J =* 6.0 Hz), 3.88 (s, 3H), 3.52 (t, 4H, *J =* 5.5 Hz), 2.78 (t, 2H, *J =* 5.5 Hz), 2.60 (t, 4H, *J =* 4.5 Hz);^13^C-NMR (DMSO-*d*_6_, 125 MHz, ppm): δ 168.9, 155.0, 153.6, 147.5, 146.8, 144.5, 141.9, 137.6, 132.8, 132.4(2C), 132.1, 131.7, 131.2, 129.6, 126.6, 125.6, 122.3(2C), 121.4, 119.1, 117.8, 116.8, 113.4, 110.2, 70.9, 57.8, 56.6, 52.9(2C), 44.5(2C); ESIMS: *m/z* 769 [M + H]^+^ HRESIMS: calc. for C_32_H_30_N_6_O_5_SBr_2_ [M + H]^+^ 769.0438, found 769.0457.

*(E)-3-(3-Bromo-5-methoxy-4-(3-(4-(pyrazin-2-yl)piperazin-1-yl)propoxy)benzylidene)-N-(4-bromophenyl)-2-oxoindoline-5-sulfonamide* (**23b**). Intermediate **14b** was treated with compound **3** following the general procedure to give the desired product **23b**. Yield: 64.1%; mp.: 124–130 °C; ^1^H-NMR (DMSO-*d*_6_, 500 MHz, ppm): δ 11.08 (s, 1H), 8.27 (s, 1H), 8.04 (s, 1H), 7.80 (s, 1H), 7.64 (s, 1H), 7.60 (s, 1H), 7.50 (s, 1H), 7.43 (s, 1H), 7.39 (d, 1H, *J =* 9.0 Hz), 7.31 (d, 2H, *J =* 8.5 Hz), 7.06 (d, 1H, *J =* 9.0 Hz), 6.96 (d, 2H, *J =* 8.5 Hz), 4.11 (t, 2H, *J =* 6.0 Hz), 3.87 (s, 3H), 3.52 (t, 4H, *J =* 5.5 Hz), 2.45-2.56 (overlap, 6H), 1.93 (m, 2H); ^13^C-NMR (DMSO-*d*_6_, 125 MHz, ppm): δ 168.9, 155.0, 153.7, 147.5, 146.8, 144.5, 141.9, 137.6, 132.8, 132.4(2C), 132.3, 131.7, 131.2, 129.6, 126.6, 125.3, 122.3(2C), 121.3, 119.1, 117.8, 116.5, 113.4, 110.8, 71.9, 56.6, 54.8, 52.8(2C), 44.5(2C), 27.6; ESIMS: *m/z* 783 [M + H]^+^ HRESIMS: calc. for C_33_H_32_N_6_O_5_SBr_2_ [M + H]^+^ 783.0594 found 783.0634.

*(E)-3-(4-(2-(Bis(2-hydroxyethyl)amino)ethoxy)-3-bromo-5-methoxybenzylidene)-N-(4-bromophenyl)-2-oxoindoline-5-sulfonamide* (**24**). Intermediate **15** was treated with compound **3** following the general procedure to give the desired product **24**. Yield: 53.4%; mp.: 135–140 °C; ^1^H-NMR (DMSO-*d*_6_, 500 MHz, ppm): δ 11.08 (s, 1H), 7.58–7.63 (overlap, 2H), 7.50 (s, 1H), 7.44 (s, 1H), 7.37 (d, 1H, *J =* 8.5 Hz), 7.28 (d, 2H, *J =* 8.5 Hz), 7.03 (d, 1H, *J =* 8.5 Hz), 6.92 (d, 2H, *J =* 8.5 Hz), 4.08 (t, 2H, *J =* 5.0 Hz), 3.88 (s, 3H), 3.44 (m, 4H), 2.67 (t, 2H, *J =* 5.0 Hz), 2.48 (m, 4H); ^13^C-NMR (DMSO-*d*_6_, 125 MHz, ppm): δ 168.9, 153.5, 147.6, 146.8, 144.3, 138.1, 132.3(2C), 132.2, 131.2, 129.6, 126.7, 125.5, 122.4(2C), 121.3, 119.1, 117.7, 116.8, 113.4, 110.7, 71.9, 59.8(2C), 57.5(2C), 56.6, 54.8; ESIMS: *m/z* 710 [M + H]^+^ HRESIMS: calc. for C_28_H_29_N_3_O_7_SBr_2_ [M + H]^+^ 710.0166, found 710.0222.

(*E)-3-(3-Bromo-4-(2-(4-(2-(dimethylamino)ethyl)piperazin-1-yl)ethoxy)-5-methoxybenzylidene)-N-(4-bromophenyl)-2-oxoindoline-5-sulfonamide* (**25**). Intermediate **16** was treated with compound **3** following the general procedure to give the desired product **25**. Yield: 50.5%; mp.: 125–130 °C; ^1^H-NMR (DMSO-*d*_6_, 500 MHz, ppm): δ 11.08 (s, 1H), 7.63 (s, 1H), 7.60 (s, 1H), 7.50 (s, 1H), 7.42 (s, 1H), 7.39 (d, 1H, *J* = 9.0 Hz), 7.30 (d, 2H, *J* = 8.5 Hz), 7.05 (d, 1H, *J* = 9.0 Hz), 6.95 (d, 2H, *J* = 8.5 Hz), 4.14 (t, 2H, *J* = 6.0 Hz), 3.86 (s, 3H), 2.70 (t, 2H, *J* = 6.0 Hz), 2.48 (m, 4H), 2.34 (m, 8H), 2.14 (s, 6H); ^13^C-NMR (DMSO-*d*_6_, 125 MHz, ppm): δ 168.9, 153.6, 147.6, 146.6, 144.4, 138.0, 132.4(2C), 132.0, 131.2, 129.7, 126.5, 125.2, 122.3(2C), 121.3, 119.1, 117.8, 116.8, 113.3, 110.8, 70.9, 57.9(2C), 56.6, 56.5, 56.0, 53.5, 53.4(2C), 45.7(2C); ESIMS: *m/z* 762 [M + H]^+^ HRESIMS: calc. for C_32_H_37_N_5_O_5_SBr_2_ [M + H]^+^ 762.0955, found 762.1093.

### 3.2. Biological Activity

#### 3.2.1. Cell Culture

A549 (human lung cancer cell line), HepG2 (human hepatocellular carcinoma cell line), Bel7402 (human hepatocellular carcinoma cell line), HCT116 (human colorectal cancer cell line), Caco2 (Human colonic epithelial cell line) were obtained from Shanghai Institute of Cell Biology and were cultured in RPMI 1640 or DMEM medium (Gibco, Big Cabin, OK, USA), supplemented with 10% fetal bovine serum (Hyclone, Logan, UT, USA), in 25 cm^2^ culture flasks at 37 °C in a humidified atmosphere with 5% CO_2_. Sunitinib was used as a reference compound.

#### 3.2.2. MTT Assay

The cell viability was assessed by MTT assay as described previously [7]. Briefly, the cells were plated at a density of 3 × 10^3^ cells/well in 96-well plates and incubated at 37 °C overnight before drug exposure. Cells were incubated with the tested compounds to achieve final concentrations (0, 2.5, 5, 10, 20, 40, 80 µg/mL) for 48 h. Twenty microliters of MTT (5 mg/mL) was added to each well and allowed to react for another 4 h. After removing the supernatant, 150 μL of DMSO was added to dissolve the formazan and the plates were read at 490 nm. All experiments were carried out in triplicate and the viability of the control cells was taken as 100% cell survival. The IC_50_ values were analyzed by GraphPad Prime 5.0.

#### 3.2.3. Colony Forming Assay

A549 cells (500 cells/well) were seeded in six-well plates. After 24 h, cells were treated with various concentrations of compound **17a** (0, 5, 10, and 20 µg/mL) and incubated for 15 days to allow colony formation. The cells were then washed with PBS, fixed with 4% paraformalclehyde for 10 min. Next, colonies were stained with 0.1% crystal violet for 15 min at room temperature. The excel crystal violet solution was washed away with distilled H_2_O, colonies containing more than 50 cells were counted and evaluated [23].

#### 3.2.4. Cell Cycle Analysis

The cell cycle was determined by flow cytometry with DNA staining to reveal the total amount of DNA. Briefly, A549 cells were seeded into six-well plates at a density of 5 × 10^5^ cells per well. The cells were treated with 5, 10 and 20 μg/mL compound **17a** for 48 h. Then the cells were harvested, washed twice with cold PBS, and fixed in cold 75% ethanol at −20 °C overnight. The next day, the cells were washed twice with cold PBS, re-suspended with cold PBS and stained with 20 µg/mL RnaseA (Sigma, St. Louis, MO, USA) for 30 min at 37 °C. The cells were washed and re-suspended with cold PBS containing 50 µg/mL propidium iodide (PI, Sigma, St. Louis, MO, USA) for 30 min at room temperature in the dark. The cell cycle phase distribution was analyzed in three different experiments using flow cytometry (Becton Dickinson, Franklin Lakes, NJ, USA).

#### 3.2.5. Cell Apoptosis Analysis

A549 cells (2 × 10^5^/well) were plated in six-well plates and incubated for 24 h, then treated with test compounds with various concentration compound **17a** (0, 5, 10, 20 µg/mL) for 48 h. Then 1~5 × 10^5^ cells were harvested, washed twice with cold PBS, re-suspended in 500 μL Annexin V Binding Buffer, and 5 μL Annexin V-FITC and 5 μL PI were added. After being stained in the dark for 10min at room temperature, the cells were analyzed in three different experiments using flow cytometry (Becton Dickinson, Franklin Lakes, NJ, USA).

#### 3.2.6. Apoptosis Assessment by Hoechst 33258 Staining

Cell morphology was evaluated by fluorescence microscopy following Hoechst 33258 staining [9]. A549 cells were cultured in six-well plates, containing 10% fetal bovine serum in the absence or presence of compound **17a** (0, 5, 10, 20 µg/mL). After 48 h incubation, the plates were washed twice with PBS and stained with Hoechst 33258 (Sigma, St. Louis, MO, USA) for 15 min in the dark. After being washed twice with PBS, the cells were visualized under a fluorescence microscope.

#### 3.2.7. Reactive Oxygen Species (ROS) Levels Assay

The production of ROS was monitored by flow cytometry using 2′,7′-dichlorohydrofluorescein (DCFH-DA) (Beyotime Biotechnolog, Shanghai, China). Briefly, A549 cells were plated in six-well plates and incubated for 24 h; then, cells were treated with compound **17a** at various concentrations (0, 5, 10, and 20 µg/mL) for 48 h. Ten micromolar DCFH-DA was added to the plates and incubated for 30 min. After incubation, cells were washed twice with ice cold PBS and harvested. Cells were analyzed by flow cytometer (Becton Dickinson, Franklin Lakes, NJ, USA) and fluorescence microscope (Olympus, Tokyo, Japan).

#### 3.2.8. Western Blot Analysis

A549 cells were treated with compound **17a** for various times. Then, cells were harvested in lysis buffer RIPA for whole-cell lysates, and then incubated on ice for 15 min. The homogenates were centrifuged at 12,000 rpm for 15 min, and the supernatant was used for Western blot analysis. The protein concentration of the supernatant was determined by BCA (Beyotime Biotechnolog, Nantong, China) assay. Supernatants were mixed with SDS–PAGE sample buffer and boiled for 5 min. Samples were subsequently loaded into each lane of a 10% SDS–PAGE and transferred onto PVDF membranes (Bio-Rad, Philadelphia, PA, USA). Membranes were blocked for 1 h in 5% (*w/v*) non-fat milk and then incubated with a primary antibody at room temperature for 1 h. The following antibodies were used: Bcl-2 (Cell Signaling), Bax (Cell Signaling), PARP (Cell Signaling), caspase-3 (Cell Signaling), cyclin D1 (Cell Signaling), CDK4 (Cell Signaling), β-actin (Sangon Biotech). Having been washed 3 times in TBST, the membranes were incubated with horseradish peroxidase (HRP)-conjugated rabbit IgG (diluted 1:10,000) for 1 h at room temperature, and then washed 3 times. Finally, proteins were detected using an enhanced chemiluminescence system BeyoECL Plus (Beyotime). The load protein was normalized to β-actin. All experiments were performed in triplicate.

## 4. Conclusions

In summary, our design and synthesis have led to a series of novel anticancer agents by attaching of N-containing heterocyclic moieties to bromophenol. Among them, thirteen compounds (**17a**, **17b**, **18a**, **19a**, **19b**, **20a**, **20b**, **21a**, **21b**, **22a**, **22b**, **23a**, and **23b**) exhibited significant inhibitory activity against the tested cancer cell lines. The SARs of bromophenol derivatives were discussed: the chain length and types of the moieties of incorporation could affect the anticancer activities. The promising candidate compound **17a** could induce cell cycle arrest at G0/G1 phase and induce apoptosis in A549 cells, as well as caused DNA fragmentations, morphological changes and ROS generation. Furthermore, compound **17a** suppression of Bcl-2 levels in A549 cells induced cells apoptosis in vitro through the ROS-mediated apoptotic pathway. These results might be useful for bromophenol derivatives to be explored and developed as novel anticancer drugs. In the future, we will further investigate that compound **17a** was developed as anticancer agents by more experiments.

## Figures and Tables

**Table 1:**
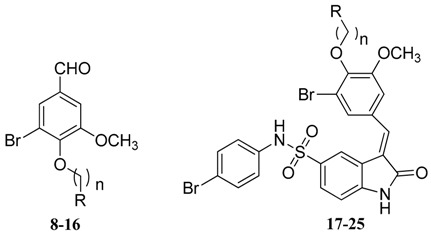
Structures of synthesized compounds **8**–**25**.

Compd.	n	R
**8a, 17a**	2	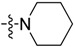
**8b, 17b**	3	
**9a, 18a**	2	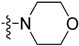
**9b, 18b**	3	
**9c, 18c**	4	
**10a, 19a**	2	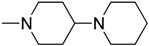
**10b, 19b**	3	
**11a, 20a**	2	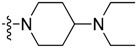
**11b, 20b**	3	
**12a, 21a**	2	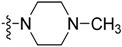
**12b, 21b**	3	
**12c, 21c**	4	
**13a, 22a**	2	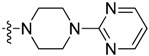
**13b, 22b**	3	
**14a, 23a**	2	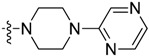
**14b, 23b**	3	
**15, 24**	2	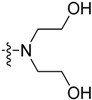
**16, 25**	2	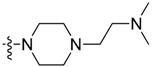

**Table 2:** Calculated physicochemical properties and predicted toxicity of compounds **17**–**25**.

Compd.	CLogP ^a^	CLogS ^b^	TPSA (Å^2^) ^c^	Ha ^d^	Hd ^e^	Toxicity Risks ^f^	Drug-Likeness ^g^
						M/T/I/R	
**17a**	4.71	−6.79	105.4	8	2	N/N/N/H	4.44
**17b**	5.16	−7.06	105.4	8	2	N/N/N/H	3.63
**18a**	3.54	−5.90	114.6	9	2	N/N/N/H	4.80
**18b**	4.00	−6.17	114.6	9	2	N/N/N/H	4.51
**18c**	4.45	−6.44	114.6	9	2	N/N/N/H	1.00
**19a**	5.21	−6.97	108.6	9	2	N/N/N/H	5.95
**19b**	5.66	−7.24	108.6	9	2	N/N/N/H	5.96
**20a**	4.89	−6.62	108.6	9	2	N/N/N/H	8.88
**20b**	5.34	−6.89	108.6	9	2	N/N/N/H	8.91
**21a**	3.65	−5.40	108.6	9	2	N/N/N/H	9.42
**21b**	4.11	−5.67	108.6	9	2	N/N/N/H	9.84
**21c**	4.56	−5.94	108.6	9	2	N/N/N/H	6.56
**22a**	3.95	−6.51	134.4	11	2	N/N/N/H	8.98
**22b**	4.41	−6.78	134.4	11	2	N/N/N/H	9.85
**23a**	3.50	−6.18	134.4	11	2	N/N/N/H	7.58
**23b**	3.95	−6.44	134.4	11	2	N/N/N/H	8.23
**24**	2.53	−5.42	145.81	10	4	H/H/L/H	3.32
**25**	3.35	−4.82	111.83	10	2	N/N/N/H	9.50

^a^ cLogP, calculated logarithm of partition coefficient between n-octanol and water; ^b^ cLogS, S: water solubility in mol/L, pH = 7.5, 25 °C; ^c^ TPSA, topological polar surface area; ^d^ Ha, hydrogen bond acceptor; ^e^ Hd, hydrogen bond donor; ^f^ Toxicity risks: M, mutagenic effect; T, tumorigenic effect; I, irritating effect; R, reproductive effect; N, none; L, low; M, medium; H, high; ^g^ The approach is based on a list of about 5300 distinct substructure fragments with associated dårug-likeness scores.

**Table 3:** IC_50_ values of bromophenol derivatives against five human cancer cell lines.

Compd.	IC_50_ (μg/mL) ^a^				
	A549	Bel7402	HepG2	HCT116	Caco2
TTEDM ^b^	NA ^c^	44.8 ± 1.45	NA	31.2 ± 1.56	25.6 ± 0.78
**wlj-18**	7.10 ± 0.53	9.68 ± 0.76	14.1 ± 1.35	9.78 ± 0.29	9.11 ± 1.23
**17a**	3.15 ± 0.43	6.10 ± 0.78	4.42 ± 0.72	5.74 ± 0.26	4.23 ± 0.32
**17b**	4.78 ± 0.56	9.98 ± 1.57	9.60 ± 0.34	3.78 ± 0.91	4.70 ± 0.35
**18a**	10.8 ± 1.21	11.8 ± 1.23	6.63 ± 0.75	6.22 ± 0.23	5.61 ± 0.89
**18b**	7.62 ± 0.76	48.6 ± 3.23	25.0 ± 1.66	16.8 ± 0.65	10.2 ± 1.28
**18c**	4.49 ± 0.73	NA	42.4 ± 1.86	NA	NA
**19a**	12.6 ± 0.98	10.9 ± 0.77	8.79 ± 0.65	4.89 ± 0.35	5.76 ± 0.82
**19b**	3.60 ± 0.38	6.78 ± 0.76	5.42 ± 0.92	3.82 ± 0.43	1.70 ± 0.24
**20a**	8.10 ± 1.11	9.40 ± 0.88	8.42 ± 0.76	5.07 ± 0.69	5.94 ± 1.32
**20b**	6.99 ± 1.42	8.67 ± 0.37	7.83 ± 1.56	5.80 ± 0.21	8.24 ± 1.42
**21a**	11.5 ± 0.27	15.1 ± 1.87	8.67 ± 0.53	5.37 ± 0.79	6.89 ± 0.55
**21b**	5.20 ± 0.76	3.25 ± 0.32	5.83 ± 1.11	4.43 ± 0.53	7.52 ± 0.99
**21c**	16.7 ± 3.39	NA	40.7 ± 2.13	43.3 ± 0.23	41.9 ± 3.89
**22a**	7.40 ± 0.46	17.3 ± 1.98	6.80 ± 1.11	3.59 ± 0.25	4.09 ± 0.76
**22b**	7.10 ± 1.02	44.8 ± 2.44	41.1 ± 3.12	34.2 ± 2.23	5.60 ± 0.46
**23a**	14.9 ± 3.26	33.7 ± 2.22	21.6 ± 1.73	8.90 ± 0.43	8.50 ± 1.81
**23b**	5.96 ± 1.02	NA	NA	22.3 ± 2.18	8.97 ± 0.29
**24**	29.9 ± 2.72	32.7 ± 0.24	NA	15.0 ± 1.36	22.7 ± 2.11
**25**	11.5 ± 2.41	12.9 ± 1.38	14.9 ± 0.87	10.1 ± 0.81	9.3 ± 1.43
Sunitinib ^d^	11.6 ± 1.24	4.50 ± 0.58	5.80 ± 1.08	3.70 ± 0.27	2.7 ± 0.35

^a^ IC_50_: Concentration of the compound producing 50% cell growth inhibition after 48 h of drug exposure, as determined by the MTT assay. Each experiment was run at least three times, and the results are presented as average values ± standard deviation; ^b^ 2,2′,3-tribromo-3′,4,4′,5-tetrahydroxy-6′-ethyloxymethyldiphenylmethane (TTEDM) is a marine bromophenol compound derived from marine *algae. P*.; ^c^ NA: Compound showing IC_50_ value > 50 μg/mL; ^d^ Sunitinib as the positive control.

## Supplementary Materials

The following are available online at www.mdpi.com/1660-3397/15/11/343/s1, Details of synthesis of intemediates **5**–**7**, HPLC Characterization of purity for the promising candidate compound **17a**.
