# Supplementary material for: Discovery of Novel Bromophenol Hybrids as Potential Anticancer Agents through the Ros-Mediated Apoptotic Pathway: Design, Synthesis and Biological Evaluation
Source: Mar Drugs. 2017 Nov 1;15(11):343. doi: 10.3390/md15110343 (PMC5706033; doi:10.3390/md15110343)
Supplement: Supplementary file 1 [file marinedrugs-15-00343-s001.pdf]

Supplementary Material

Discovery of novel bromophenol hybrids as potential anticancer agents through the ROS-mediated apoptotic pathway: design, synthesis and biological evaluation

1. Materials and instruments

Reaction reagents were purchased from J&K Scientific Ltd. Organic solvents were analytical reagent grade and purchased from Tianjin Chemical Reagent Co., Ltd. Column chromatography (CC): silica gel (200–300 mesh; Qingdao Makall Group Co., Ltd; Qingdao; China). All reactions were monitored using thin-layer chromatography (TLC) on silica gel plates. ^{1}H and ^{13}C NMR spectra were recorded on Bruker DRX 500 MHz spectrometers with tetramethylsilane (TMS) as the internal standard (Bruker, Bremerhaven, Germany). MS and HRMS spectra were determined on a LCMS–IT–TOF mass spectrometer (Shimadzu, Kyoto, Japan). Melting points were determined on a SGW X-4 Melting Point Apparatus (Shanghai Precision Science Instrument Co., Ltd; Shanghai; China). The synthesized compounds were named using ChemBioDraw Ultra software (v 12.0).

2. Synthesis and structure data of intemediates (5-16)

2.1. General procedures for the preparation of compounds ***5***-***7***

The mixture of anhydrous K_2CO_3 (1.00 eq.) and the 3-bromo-4-hydroxy-5-methoxybenzaldehyde **4** (1.00 eq.) were suspended in dry DMF. Then an alkyl dibromide (1.10 eq.) was added in several portions (neat) during 0.5 h. This reaction mixture was stirred at 60-80°C over night. After complete turnover water was added and the aqueous solution was extracted three times with chloroform. The combined organic layers and washed with water, HCl and brine. And then the organic layer was dried with Na_2SO_4 and removed in vacuo. The resulting residue was purified by column chromatography on silica gel to provide the compounds **5**-**7**.

*2.1.1 3-Bromo-4-(2-bromoethoxy)-5-methoxybenzaldehyde (**5**)*

Compound **4** was treated with 1, 2-dibromoethane according to general procedure to give compound **5** as a white solid. Yield: 79.1%; ^{1}H NMR ($CDCl_3$, 500 MHz, ppm): δ 9.82(s, 1H), 7.63(s, 1H), 7.37(s, 1H), 4.39(t, 2H, J= 7.0 Hz), 3.92(s, 3H), 3.65(t, 2H, J= 7.0 Hz); ^{13}C NMR ($CDCl_3$, 125MHz, ppm): δ 189.7, 153.8, 150.0, 133.2, 128.7, 118.0, 110.1, 72.7, 56.3, 29.3.

*2.1.2 3-Bromo-4-(3-bromopropoxy)-5-methoxybenzaldehyde (**6**)*

Compound **4** was treated with 1,3-dibromopropane according to general procedure to give compound **6** as colorless oil. Yield: 73.4%; ^{1}H NMR ($CDCl_3$, 500 MHz, ppm): δ 9.77(s, 1H), 7.57(s, 1H), 7.31(s, 1H), 4.18(t, 2H, J= 6.0 Hz), 3.87(s, 3H), 3.67(t, 2H, J= 6.5 Hz), 2.27(m, 2H); ^{13}C NMR ($CDCl_3$, 125MHz, ppm): δ 189.7, 153.9, 150.5, 133.0, 128.5, 117.9, 110.1, 70.9, 56.2, 33.4, 30.2.

2.1.3 3-Bromo-4-(4-bromobutoxy)-5-methoxybenzaldehyde (7)

Compound **4** was treated with 1,4-dibromobutane according to general procedure to give compound **7** as colorless oil. Yield: 76.7%; ^{1}H NMR ($CDCl_3$, 500 MHz, ppm): δ 9.81(s, 1H), 7.62(s, 1H), 7.35(s, 1H), 4.11(t, 2H, J= 6.0 Hz), 3.90(s, 3H), 3.52(t, 2H, J= 6.5 Hz), 2.14(m, 2H), 1.94(m, 2H); ^{13}C NMR ($CDCl_3$, 125MHz, ppm): δ 189.8, 154.1, 150.9, 132.9, 128.7, 118.0, 110.1, 72.4, 56.2, 33.5, 29.3, 28.7.

*2.2 General procedure for the preparation of compounds **8-16***

To a solution of compounds **5**–**7** (0.1 mmol) in DMF was added the appropriate amine (0.3 mmol) and Et_3N (160 μL, 1.17 mmol). After stirring at room temperature (unless otherwise indicated) for overnight, the mixture was poured into water. Then, the organic phase was extracted three times with EtOAc (4 × 30 mL) and washed with H2O (2 × 30 mL) and with brine (2 × 30 mL). The solvent was evaporated, and the residue was purified by column chromatography on silica gel to provide the compounds **8-16**.

*2.2.1 3-Bromo-5-methoxy-4-(2-(piperidin-1-yl)ethoxy)benzaldehyde (**8a**)*

Intermediate **5** was treated with piperidine following the general procedure to give the desired product **8a** as white solid. Yield: 77.8%; ^{1}H NMR ($CDCl_3$, 500 MHz, ppm): δ 9.79(s, 1H), 7.60(s, 1H), 7.32(s, 1H), 4.21(t, 2H, J= 6.0 Hz), 3.87(s, 3H), 2.78(t, 2H, J= 6.0 Hz), 2.48(m, 4H), 1.54(m, 4H), 1.39(m, 2H); ^{13}C NMR ($CDCl_3$, 125MHz, ppm): δ 189.8, 154.0, 151.2, 132.8, 128.9, 118.1, 109.9, 70.7, 58.6, 56.2, 54.8 (2C), 25.8 (2C), 24.2.

*2.2.2 3-Bromo-5-methoxy-4-(3-(piperidin-1-yl)propoxy)benzaldehyde (**8b**)*

Intermediate **6** was treated with piperidine following the general procedure to give the desired product **8b** as white solid. Yield: 81.6%; ^{1}H NMR ($CDCl_3$, 500 MHz, ppm): δ 9.82(s, 1H), 7.64(s, 1H), 7.36(s, 1H), 4.16(t, 2H, J= 6.0 Hz), 3.90(s, 3H), 2.56(t, 2H, J= 7.5 Hz), 2.43(m, 4H), 2.00(m, 2H, J= 7.0 Hz), 1.59(m, 4H), 1.44(m, 2H); ^{13}C NMR ($CDCl_3$, 125MHz, ppm): δ 189.8, 154.1, 151.2, 132.8, 128.8, 118.1, 110.0, 72.4, 56.2, 55.9, 54.6 (2C), 27.6, 25.9 (2C), 24.4.

*2.2.3 3-Bromo-5-methoxy-4-(4-(piperidin-1-yl)butoxy)benzaldehyde (**8c**)*

Intermediate **7** was treated with piperidine following the general procedure to give the desired product **8c** as solid. Yield: 82.3%; ^{1}H NMR ($CDCl_3$, 500 MHz, ppm): δ 9.82(s, 1H), 7.64(s, 1H), 7.36(s, 1H), 4.16(t, 2H, *J*= 6.0 Hz), 3.90(s, 3H), 2.56(t, 2H, *J*= 7.5 Hz), 2.43(m, 4H), 2.00(m, 2H, *J*= 7.0 Hz), 1.59(m, 4H), 1.44(m, 2H); ^{13}C NMR ($CDCl_3$, 125MHz, ppm): δ 189.8, 154.1, 151.2, 132.8, 128.8, 118.1, 110.0, 72.4, 56.2, 55.9, 54.6 (2C), 27.6, 25.9 (2C), 24.4.

*2.2.4 3-Bromo-5-methoxy-4-(2-morpholinoethoxy)benzaldehyde (**9a**)*

Intermediate **5** was treated with morpholine following the general procedure to give the desired product **9a** as solid. Yield: 81.1%; ^{1}H NMR ($CDCl_3$, 500 MHz, ppm): δ 9.82(s, 1H), 7.64(s, 1H), 7.36(s, 1H), 4.22(t, 2H, *J*= 5.5 Hz), 3.90(s, 3H), 3.70(t, 4H, *J*= 4.5 Hz), 2.82(t, 2H, *J*= 5.5 Hz), 2.57(t, 4H, *J*= 4.5 Hz); ^{13}C NMR ($CDCl_3$, 125MHz, ppm): δ 189.8, 154.1, 151.0, 133.0, 128.8, 118.1, 109.9, 70.3, 66.9(2C), 58.4, 56.2, 53.9(2C).

*2.2.5 3-Bromo-5-methoxy-4-(3-morpholinopropoxy)benzaldehyde (**9b**)*

Intermediate **6** was treated with morpholine following the general procedure to give the desired product **9b** as solid. Yield: 71.3%;^{1}H NMR ($CDCl_3$, 500 MHz, ppm): δ 9.74(s, 1H), 7.55(s, 1H), 7.28(s, 1H), 4.08(t, 2H, *J*= 6.0 Hz), 3.82(s, 3H), 3.61(t, 4H, *J*= 4.5 Hz), 2.50(t, 2H, *J*= 7.0 Hz), 2.38(m, 4H), 1.90(m, 2H); ^{13}C NMR ($CDCl_3$, 125MHz, ppm): δ 189.8, 154.0, 150.9, 132.8, 128.5, 117.9, 110.2, 71.9, 66.8(2C), 56.1, 55.3, 53.6(2C), 27.2.

*2.2.6 3-Bromo-5-methoxy-4-(4-morpholinobutoxy)benzaldehyde (**9c**)*

Intermediate **7** was treated with morpholine following the general procedure to give the desired product **9c** as solid. Yield: 68.2%; ^{1}H NMR ($CDCl_3$, 500 MHz, ppm): δ 9.83(s, 1H), 7.64(s, 1H), 7.37(s, 1H), 4.11(t, 2H, *J*= 6.0 Hz), 3.91(s, 3H), 3.71(t, 4H, *J*= 4.5 Hz), 2.39-2.44(overlap, 6H), 1.84(m, 2H), 1.73(m, 2H); ^{13}C NMR ($CDCl_3$, 125MHz, ppm): δ 189.8, 154.1, 151.2, 132.8, 128.9, 118.1, 110.0, 73.5, 67.0(2C), 58.7, 56.2, 53.7(2C), 28.2, 23.0.

*2.2.7 4-(2-([1,4'-bipiperidin]-1'-yl)ethoxy)-3-bromo-5-methoxybenzaldehyde (**10a**)*

Intermediate **5** was treated with 1,4'-bipiperidine following the general procedure to give the desired product **10a** as solid. Yield: 66.9%; ^{1}H NMR ($CDCl_3$, 500 MHz, ppm): δ 9.81(s, 1H), 7.62(s, 1H), 7.34(s, 1H), 4.09(t, 2H, *J*= 6.0 Hz), 3.89(s, 3H), 2.79(t, 2H, *J*= 6.0 Hz), 2.44-2.48(overlap, 9H), 1.54(m, 8H), 1.40(m, 2H); ^{13}C NMR ($CDCl_3$, 125MHz, ppm): δ 189.9, 153.0, 144.6, 133.2, 128.8, 116.9, 109.9, 70.8, 62.8, 57.9, 56.1, 53.9 (2C), 50.1(2C), 27.7(2C), 26.4 (2C), 24.9.

*2.2.8 4-(3-([1,4'-bipiperidin]-1'-yl)propoxy)-3-bromo-5-methoxybenzaldehyde (**10b**)*

Intermediate **6** was treated with 1,4'-bipiperidine following the general procedure to give the desired product **10b** as solid. Yield: 73.2%; ^{1}H NMR ($CDCl_3$, 500 MHz, ppm): δ 9.78(s, 1H), 7.59(s, 1H), 7.32(s, 1H), 4.11(t, 2H, J= 6.0 Hz), 3.86(s, 3H), 2.95(t, 2H, J= 6.0 Hz), 2.48-2.53(overlap, 6H), 2.25(m, 1H), 1.89-1.97(overlap, 4H), 1.75(m, 2H), 1.55(m, 6H), 1.38(m, 2H); ^{13}C NMR ($CDCl_3$, 125MHz, ppm): δ 189.8, 154.0, 151.1, 132.7, 128.7, 118.0, 110.0, 72.3, 62.8, 56.1, 55.0, 53.5 (2C), 50.1(2C), 27.9, 27.7(2C), 26.3 (2C), 24.7.

*2.2.9 4-(4-([1,4'-bipiperidin]-1'-yl)butoxy)-3-bromo-5-methoxybenzaldehyde (**10c**)*

Intermediate **7** was treated with 1,4'-bipiperidine following the general procedure to give the desired product **10c** as solid. Yield: 68.2%; ^{1}H NMR ($CDCl_3$, 500 MHz, ppm): δ 9.80(s, 1H), 7.53(s, 1H), 7.33(s, 1H), 4.08(t, 2H, J= 6.0 Hz), 3.87(s, 3H), 2.97(t, 2H, J= 6.0 Hz), 2.34-2.37(overlap, 6H), 2.05(m, 1H), 1.90(m, 4H), 1.49-1.79(overlap, 10H) 1.39(m, 2H); ^{13}C NMR ($CDCl_3$, 125MHz, ppm): δ 189.8, 154.1, 151.1, 132.7, 128.7, 118.1, 110.1, 73.6, 62.8, 56.2, 55.6, 53.5 (2C), 50.4(2C), 27.5, 26.1, 26.0(2C), 24.6 (2C), 24.5.

*2.2.10 3-bromo-4-(2-(4-(diethylamino)piperidin-1-yl)ethoxy)-5-methoxybenzaldehyde (**11a**)*

Intermediate **5** was treated with N, N-diethylpiperidin-4-amine following the general procedure to give the desired product **11a** as solid. Yield: 72.5%; ^{1}H NMR ($CDCl_3$, 500 MHz, ppm): δ 9.80(s, 1H), 7.62(s, 1H), 7.34(s, 1H), 4.20(t, 2H, J= 6.0 Hz), 3.89(s, 3H), 3.05(d, 2H, J= 11.5 Hz), 2.78(t, 2H, J= 6.0 Hz), 2.51-2.56(overlap, 5H), 2.04 (t, 2H, J= 6.0 Hz), 1.71(d, 2H, J= 11.5 Hz), 1.53(m, 2H), 1.02(t, 6H, J= 7.0 Hz); ^{13}C NMR ($CDCl_3$, 125MHz, ppm): δ 189.8, 154.0, 151.2, 132.8, 128.7, 118.1, 109.9, 70.8, 58.1, 57.9, 56.2, 53.8 (2C), 43.6(2C), 28.2(2C), 13.6 (2C).

*2.2.11 3-bromo-4-(3-(4-(diethylamino)piperidin-1-yl)propoxy)-5-methoxybenzaldehyde (**11b**)*

Intermediate **6** was treated with N,N-diethylpiperidin-4-amine following the general procedure to give the desired product **11b** as solid. Yield: 76.3%; ^{1}H NMR ($CDCl_3$, 500 MHz, ppm): δ 9.82(s, 1H), 7.63(s, 1H), 7.35(s, 1H), 4.15(t, 2H, J= 6.0 Hz), 3.90(s, 3H), 3.00(d, 2H, J= 11.0 Hz), 2.54-2.60(overlap, 7H), 1.93-2.00 (m, 4H), 1.75(d, 2H, J= 11.0 Hz), 1.58(m, 2H), 1.04(t, 6H, J= 7.0 Hz); ^{13}C NMR ($CDCl_3$, 125MHz, ppm): δ 189.8, 154.1, 151.1, 132.8, 128.8, 118.0, 110.0, 72.3, 58.2, 56.2, 55.0, 53.6 (2C), 43.6(2C), 28.3(2C), 27.9, 13.5(2C).

*2.2.12 3-bromo-4-(4-(4-(diethylamino)piperidin-1-yl)butoxy)-5-methoxybenzaldehyde (**11c**)*

Intermediate **7** was treated with N, N-diethylpiperidin-4-amine following the general procedure to give the desired product **11c** as solid. Yield: 63.7%; ^{1}H NMR ($CDCl_3$, 500 MHz, ppm): δ 9.80(s, 1H),

7.61(s, 1H), 7.33(s, 1H), 4.08(t, 2H, *J*= 6.0 Hz), 3.88(s, 3H), 3.00(d, 2H, *J*= 11.0 Hz), 2.37-2.59(overlap, 7H), 1.88-2.00 (m, 4H), 1.54-1.80(overlap, 6H), 1.03(t, 6H, *J*= 7.0 Hz); ^{13}C NMR ($CDCl_3$, 125MHz, ppm): δ 189.9, 153.9, 151.1, 132.6, 128.7, 118.0, 110.4, 73.4, 57.7, 56.0, 55.5, 53.4 (2C), 43.5(2C), 28.3(2C), 27.9, 25.2, 13.3(2C).

*2.2.13 3-bromo-5-methoxy-4-(2-(4-methylpiperazin-1-yl)ethoxy)benzaldehyde (**12a**)*

Intermediate **5** was treated with 1-methylpiperazine following the general procedure to give the desired product **12a** as solid. Yield: 66.6%; ^{1}H NMR ($CDCl_3$, 500 MHz, ppm): δ 9.82(s, 1H), 7.63(s, 1H), 7.36(s, 1H), 4.22(t, 2H, *J*= 5.5 Hz), 3.90(s, 3H), 2.83(t, 2H, *J*= 5.5 Hz), 2.62(broad, 4H), 2.47(broad, 4H), 2.27(s, 3H); ^{13}C NMR ($CDCl_3$, 125MHz, ppm): δ 189.8, 154.1, 151.1, 132.9, 128.8, 118.1, 109.9, 70.6, 57.9, 56.2, 55.0(2C), 53.3(2C), 46.0.

*2.2.14 3-bromo-5-methoxy-4-(3-(4-methylpiperazin-1-yl)propoxy)benzaldehyde (**12b**)*

Intermediate **6** was treated with 1-methylpiperazine following the general procedure to give the desired product **12b** as solid. Yield: 71.4%; ^{1}H NMR ($CDCl_3$, 500 MHz, ppm): δ 9.82(s, 1H), 7.63(s, 1H), 7.36(s, 1H), 4.15(t, 2H, *J*= 5.5 Hz), 3.90(s, 3H), 2.62-2.47(overlap, 10H), 2.27(s, 3H), 1.81(m, 2H); ^{13}C NMR ($CDCl_3$, 125MHz, ppm): δ 189.8, 154.1, 151.2, 132.8, 128.8, 118.0, 110.0, 72.6, 58.2, 56.2, 55.1(2C), 53.1(2C), 46.0, 27.8.

*2.2.15 3-bromo-5-methoxy-4-(4-(4-methylpiperazin-1-yl)butoxy)benzaldehyde (**12c**)*

Intermediate **7** was treated with 1-methylpiperazine following the general procedure to give the desired product **12c** as solid. Yield: 71.3%; ^{1}H NMR ($CDCl_3$, 500 MHz, ppm): δ 9.82(s, 1H), 7.63(s, 1H), 7.36(s, 1H), 4.10(t, 2H, *J*= 5.5 Hz), 3.90(s, 3H), 2.62(broad, 4H), 2.47(broad, 4H), 2.41(t, 2H, *J*= 5.5 Hz), 2.27(s, 3H), 1.82(m, 2H), 1.72(m, 2H); ^{13}C NMR ($CDCl_3$, 125MHz, ppm): δ 189.8, 154.1, 151.2, 132.8, 128.8, 118.1, 110.0, 73.5, 58.2, 56.2, 55.1(2C), 53.1(2C), 46.0, 28.2, 23.3.

*2.2.16 3-bromo-5-methoxy-4-(2-(4-(pyrimidin-2-yl)piperazin-1-yl)ethoxy)benzaldehyde (**13a**)*

Intermediate **5** was treated with 2-(piperazin-1-yl)pyrimidine following the general procedure to give the desired product **13a** as solid. Yield: 72.9%; ^{1}H NMR ($CDCl_3$, 500 MHz, ppm): δ 9.66(s, 1H), 8.12(d, 2H, *J*= 4.5 Hz), 7.83(s, 1H), 7.47(s, 1H), 6.31(t, 1H, *J*= 4.5 Hz), 4.11(t, 2H, *J*= 5.5 Hz), 3.75(s, 3H), 3.65(t, 4H, *J*= 5.5 Hz), 2.71(t, 2H, *J*= 5.5 Hz), 2.47(t, 4H, *J*= 4.5 Hz); ^{13}C NMR ($CDCl_3$, 125MHz, ppm): δ 189.6, 161.5, 157.5(2C), 154.0, 150.8, 132.8, 128.3, 118.0, 110.1, 109.7, 70.3, 57.9, 56.1, 53.1(2C), 43.5(2C).

*2.2.17 3-bromo-5-methoxy-4-(3-(4-(pyrimidin-2-yl)piperazin-1-yl)propoxy)benzaldehyde (**13b**)*

Intermediate **6** was treated with 2-(piperazin-1-yl)pyrimidine following the general procedure to give the desired product **13b** as solid. Yield: 66.1%;^{1}H NMR ($CDCl_3$, 500 MHz, ppm): δ 9.76(s, 1H), 8.22(d, 2H, J= 4.5 Hz), 7.94(s, 1H), 7.58(s, 1H), 6.41(t, 1H, J= 4.5 Hz), 4.14(t, 2H, J= 5.5 Hz), 3.85(s, 3H), 3.76(t, 4H, J= 5.5 Hz), 2.58(t, 2H, J= 5.5 Hz), 2.47(t, 4H, J= 4.5 Hz), 1.98(m, 2H); ^{13}C NMR ($CDCl_3$, 125MHz, ppm): δ 189.8, 161.6, 157.8(2C), 154.0, 151.0, 132.8, 128.7, 118.0, 110.1, 109.7, 72.0, 56.2, 55.0, 53.0(2C), 43.6(2C), 27.5.

*2.2.18 3-bromo-5-methoxy-4-(4-(4-(pyrimidin-2-yl)piperazin-1-yl)butoxy)benzaldehyde (**13c**)*

Intermediate **7** was treated with 2-(piperazin-1-yl)pyrimidine following the general procedure to give the desired product **13c** as solid. Yield: 75.3%;^{1}H NMR ($CDCl_3$, 500 MHz, ppm): δ 9.80(s, 1H), 8.26(d, 2H, J= 4.5 Hz), 7.62(s, 1H), 7.34(s, 1H), 6.44(t, 1H, J= 4.5 Hz), 4.11(t, 2H, J= 5.5 Hz), 3.88(s, 3H), 3.80(t, 4H, J= 5.5 Hz), 2.49(t, 2H, J= 5.5 Hz), 2.44(t, 4H, J= 4.5 Hz), 1.84(m, 2H), 1.75(m, 2H); ^{13}C NMR ($CDCl_3$, 125MHz, ppm): δ 189.8, 161.5, 157.6(2C), 154.0, 151.0, 132.6, 128.7, 118.0, 110.1, 109.8, 73.5, 58.3, 56.2, 53.1(2C), 43.7(2C), 28.2, 23.2.

*2.2.19 3-bromo-5-methoxy-4-(2-(4-(pyrazin-2-yl)piperazin-1-yl)ethoxy)benzaldehyde (**14a**)*

Intermediate **5** was treated with 2-(piperazin-1-yl)pyrazine following the general procedure to give the desired product **14a** as white solid. Yield: 78.4%;^{1}H NMR ($CDCl_3$, 500 MHz, ppm): δ 9.78(s, 1H), 8.08(s, 1H), 8.00(s, 1H), 7.78(s, 1H), 7.60(s, 1H), 7.33(s, 1H), 4.23(t, 2H, J= 5.5 Hz), 3.87(s, 3H), 3.56(t, 4H, J= 5.5 Hz), 2.85(t, 2H, J= 5.5 Hz), 2.66(t, 4H, J= 4.5 Hz); ^{13}C NMR ($CDCl_3$, 125MHz, ppm): δ 189.7, 154.9, 154.0, 150.9, 141.7, 132.9, 132.8, 131.0, 128.9, 118.1, 110.0, 70.4, 58.0, 56.2, 52.9(2C), 44.4(2C).

*2.2.20 3-bromo-5-methoxy-4-(3-(4-(pyrazin-2-yl)piperazin-1-yl)propoxy)benzaldehyde (**14b**)*

Intermediate **6** was treated with 2-(piperazin-1-yl)pyrazine following the general procedure to give the desired product **14b** as white solid. Yield: 71.9%; ^{1}H NMR ($CDCl_3$, 500 MHz, ppm): δ 9.79(s, 1H), 8.08(s, 1H), 8.00(s, 1H), 7.78(s, 1H), 7.61(s, 1H), 7.33(s, 1H), 4.15(t, 2H, J= 5.5 Hz), 3.87(s, 3H), 3.56(t, 4H, J= 5.5 Hz), 2.62(t, 2H, J= 5.5 Hz), 2.55(t, 4H, J= 4.5 Hz), 1.99(m, 2H); ^{13}C NMR ($CDCl_3$, 125MHz, ppm): δ 189.8, 155.0, 154.0, 151.0, 141.7, 132.9, 132.8, 130.9, 128.7, 118.0, 110.1, 71.9, 56.2, 55.0, 52.7(2C), 44.4(2C), 27.5.

*2.2.21 4-(2-(bis(2-hydroxyethyl)amino)ethoxy)-3-bromo-5-methoxybenzaldehyde (**15**)*

Intermediate **5** was treated with 2,2'-azanediyldiethanol following the general procedure to give the desired product **15** as white solid. Yield: 61.3%; ^{1}H NMR ($CDCl_3$, 500 MHz, ppm): δ 9.83(s, 1H),

7.64(s, 1H), 7.37(s, 1H), 4.11(t, 2H, *J*= 5.0 Hz), 3.92(s, 3H), 3.65(t, 4H, *J*= 5.0 Hz), 3.00 (t, 2H, *J*= 5.0 Hz), 2.80(t, 4H, *J*= 5.0 Hz); ^{13}C NMR ($CDCl_3$, 125MHz, ppm): δ 189.7, 154.0, 150.4, 133.3, 128.7, 118.2, 110.1, 72.1, 59.6(2C), 57.4(2C), 56.3, 54.5.

3. HPLC Characterization of purity for the promising candidate compound 17a

Purity of compound 17a was measured by HPLC was carried out on a Shimadzu LC-20A system equipped with a Shimadzu InertSustain C-18 reverse phase column (4.6mm*250mm*5um) and SPD-20A detector.

Column tmperature: 40 ℃.

Flow rate: 1 mL/min.

Detection wavelength: 254 nm.

Run time: 30.0 minutes.

Injection volume: 20 μL.

Mobile phases A: acetonitrile and water (5: 95 – 100:0); Purity: 95.63%.

Mobile phases B: methanol and water (10: 90 – 100:0); Purity: 95.37%.

References

[1] Wang SY, Wang LJ, Jiang B, Wu N, Li XQ, Luo J, Wang BC, Zhang RS, Xu Q, Shi DY, *RSC Adv.,* 2015;5:91795-91801.

[2] Alza NP, Richmond V, Baier CJ, Freire E, Baggio R, Murray AP, *Bioorg. Med. Chem.*, 2014;22:3838-3849.

[3] Taddei M, Ferrini S, Giannotti L, Corsi M, Manetti F, Giannini G, Vesci L, Milazzo FM, Alloatti D, Guglielmi MB, Castorina M, Cervoni ML, Barbarino M, Fodera R, Carollo V, Pisano C, Armaroli S, Cabri W, *J. Med. Chem.*, 2014;57:2258-2274.

[4] Lai YS, Ma L, Huang WX, Yu X, Zhang YH, Ji H, Tian JD, *Bioorg. Med. Chem.*, 2010;20:7349-7353.

[5] Zheng GH, Shen JJ, Zhan YC, Yi H, Xue ST, Wang Z, Ji XY, Li ZR, *Eur. J.Med. Chem.*, 2014;81:277-288.
